# Broad Adaptability of Coronavirus Adhesion Revealed from the Complementary Surface Affinity of Membrane and Spikes

**DOI:** 10.1002/advs.202404186

**Published:** 2024-09-04

**Authors:** Aritz B. García‐Arribas, Pablo Ibáñez‐Freire, Diego Carlero, Pablo Palacios‐Alonso, Miguel Cantero‐Reviejo, Pablo Ares, Guillermo López‐Polín, Han Yan, Yan Wang, Soumya Sarkar, Manish Chhowalla, Hanna M. Oksanen, Jaime Martín‐Benito, Pedro J. de Pablo, Rafael Delgado‐Buscalioni

**Affiliations:** ^1^ Departamento de Física de la Materia Condensada Universidad Autónoma de Madrid Madrid 28049 Spain; ^2^ Departamento de Física Teórica de la Materia Condensada Universidad Autónoma de Madrid Madrid 28049 Spain; ^3^ Departamento de Estructura de Macromoléculas Centro Nacional de Biotecnología CSIC Madrid 28049 Spain; ^4^ Department of Materials Science and Metallurgy University of Cambridge Cambridge CB3 0FS UK; ^5^ Faculty of Biological and Environmental Sciences Vijkki Biocenter University of Helsinki Helsinki 00014 Finland; ^6^ Instituto de Física de la Materia Condensada IFIMAC Universidad Autónoma de Madrid Madrid 28049 Spain

**Keywords:** atomic‐force‐microscopy, coarse‐graining models, coronavirus, elastic theory, surface‐affinity

## Abstract

Coronavirus stands for a large family of viruses characterized by protruding spikes surrounding a lipidic membrane adorned with proteins. The present study explores the adhesion of transmissible gastroenteritis coronavirus (TGEV) particles on a variety of reference solid surfaces that emulate typical virus‐surface interactions. Atomic force microscopy informs about trapping effectivity and the shape of the virus envelope on each surface, revealing that the deformation of TGEV particles spans from 20% to 50% in diameter. Given this large deformation range, experimental Langmuir isotherms convey an unexpectedly moderate variation in the adsorption‐free energy, indicating a viral adhesion adaptability which goes beyond the membrane. The combination of an extended Helfrich theory and coarse‐grained simulations reveals that, in fact, the envelope and the spikes present complementary adsorption affinities. While strong membrane‐surface interaction lead to highly deformed TGEV particles, surfaces with strong spike attraction yield smaller deformations with similar or even larger adsorption‐free energies.

## Graphical abstract

The affinity of TGEV coronavirus and its adsorption energy on different surfaces is measured by Langmuir isotherms obtained from large AFM images and individual virus profiles. Substantial adsorption‐free energy Δ*G* is found in all surfaces, however, in contrast with vesicle adsorption, the virus deformation and Δ*G* are not strongly correlated. Elastic theory and simulations reveal this adhesion adaptability of coronavirus is due to the complementary adsorption affinity of the envelope and the viral spikes. Depending on the surface, the coronavirus adsorbs using its membrane (higher deformation) or its spikes (lower deformation).

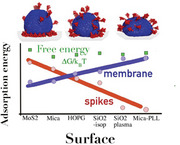

## 1 Introduction

The study of viruses is intimately connected to their interactions with surfaces. From the biological standpoint, the cell membrane is the first barrier a virus needs to cross for infection. From the prophylactic view, inanimate surfaces might act as fomites for disease dissemination,^[^ ^1^ ^]^ but also, new materials are actively being searched to maximize virus‐trapping and deactivation.^[^ ^2^ ^]^ Indeed, understanding virus‐surface interaction is extremely relevant both for biological and health concerns.^[^ ^3^ ^]^ The adhesion free energy Δ*G*, determining the virus affinity for a surface, is a thermodynamic quantity measurable via the dissociation constant *K*_*d*_, which depends on the interfacial free energy per area *W* and on mechanical properties of the viral particle.^[^ ^4^ ^]^ These features including surface tension Σ and bending rigidity κ, are essential to regulate the adhesion of enveloped virus.^[^ ^5^ ^]^ The virus particles of coronavirus (CoV) family takes its lipid envelope from the host cell, becoming essentially spherical particles containing single‐stranded positive‐sense RNA packed with nucleoprotein (N) to form the ribonucleoprotein.^[^ ^6^ ^]^ Major common features of CoV include several key membrane‐bound proteins: small envelope proteins (E), membrane proteins (M) and the spike glycoproteins (S) protruding out of the membrane. The standard picture of the initial step of infection consists of the receptor‐binding domain (RBD) at the end of the spike protein binding to the cell receptor. In this picture, the virus lipid envelope is far away and has a minor role. Yet, the CoV structure has a radius of ≈40 nm,^[^ ^7^ ^]^ and the spikes of 17 nm, are known to be translationally mobile and flexible.^[^ ^8^, ^9^ ^]^ This leaves room for interaction between the cell membrane and the virus lipid envelope which might be attractive and lead to membrane fusion.^[^ ^10^ ^]^ All these facts (further evidences given in the Discussion section) highlight the relevance of the coronavirus envelope in adhesion and infectious process, a contribution which have been largely disregarded in the literature. This work highlights the role of the lipid envelope in coronavirus adhesion on hard substrates. Yet, it is possible to extrapolate that the envelope contribution will also be important on soft substrates,^[^ ^10^, ^11^, ^12^ ^]^ and also probably on the cell membrane, a question which deserves further investigation.

The transmissible gastroenteritis virus (TGEV) is a human‐safe swine coronavirus which shares with SARS‐CoV‐2 most of the structural features.^[^ ^13^, ^14^, ^15^ ^]^ TGEV and SARS‐CoV‐2 present a similar compression rigidity or effective “spring constant” under atomic force microscopy (AFM), *k*_*sp*_ ≃ 0.01 N*m* ^−1^.^[^ ^14^, ^16^ ^]^ There is a strong correlation between the effects of different antiviral metal nanoparticles across TGEV and SARS‐CoV‐2.^[^ ^17^ ^]^ In fact, due to the strong similarities, TGEV is one of the most frequently used surrogates for SARS‐CoV,^[^ ^14^, ^18^, ^19^ ^]^ and we use it as a model system to study the adhesion of CoV structures on surfaces. Although TGEV is polymorphic it is possible to ascribe a radius of *R* = (41 ± 2) nm (value from cryo‐EM.^[^ ^14^, ^20^ ^]^) TGEV is decorated with ≈40 spikes of 15–20 nm in length^[^ ^7^, ^20^ ^]^ which are remarkably mobile and flexible.^[^ ^8^, ^9^, ^16^ ^]^ The virus envelope is mainly composed by the lipid membrane taken from the host cell, thus hydrophilic and negatively charged, however, virus proteins are also present providing hydrophobic patches. The spikes of the TGEV present a positively charged head and negatively charged patches along their sides, mostly due to the decorating glycoproteins.

The adhesion of CoV to material surfaces has been studied for virus inactivation purposes.^[^ ^1^, ^3^ ^]^ Also, comparative analyses showed that enveloped viruses adhere more to solid fraction of wastewaters than non‐enveloped ones. Moreover, coronaviruses (SARS‐CoV‐2 in that study) presented the largest adhesive strength amongst all the enveloped considered viruses, particularly at ambient temperature.^[^ ^21^ ^]^ Despite, little attention has been paid to fundamental physical quantities such as the adhesion free energy Δ*G*, envelope surface tension Σ and bending rigidity κ. The standard route to Δ*G* is the construction of Langmuir isotherms,^[^ ^22^ ^]^ which leads to the equilibrium constant or its inverse, the dissociation constant *K*_*d*_/[1M] ≈ exp (Δ*G*/*k*_*B*_ *T*). In this vein, during the past century some experimental studies on viral adsorption provided Δ*G* based on Langmuir isotherms.^[^ ^22^, ^23^, ^24^, ^25^ ^]^ Only very recently some experiments appear based on quartz‐microbalance,^[^ ^26^, ^27^ ^]^ providing qualitative information which is difficult to quantify.^[^ ^28^ ^]^ In addition, AFM and electron microscopy have been also used to study CoV adsorption on surfaces.^[^ ^29^ ^]^ Measurements of the very thermodynamic affinity or dissociation constant *K*_*d*_ of CoV on surfaces has not been yet accomplished. Interestingly, the physicochemical mechanisms regarding colloidal adsorption onto surfaces are mainly known:^[^ ^10^ ^]^ electrostatic interactions,^[^ ^23^, ^30^ ^]^ van der Waals forces,^[^ ^24^, ^25^ ^]^ hydrophobic effects,^[^ ^31^ ^]^ formation of hydrogen‐bonds,^[^ ^32^ ^]^ or dipolar interactions.^[^ ^10^ ^]^ However, when dealing with highly inhomogeneous biological analytes such as virus particles, the estimation of their relative importance remains difficult to achieve. Capsid amino acids can be positively ionized to enhance adsorption^[^ ^33^ ^]^ but this ionization can also be influenced by the inner genome negative charge.^[^ ^34^ ^]^ While virus adsorption is highly influenced by both electrostatic interactions and hydrophobic effects,^[^ ^35^ ^]^ Van der Waals interactions are relevant in neutral surfaces.^[^ ^3^ ^]^ The topic of adsorption mechanisms reaches even higher levels of complexity if we consider the variability between non‐enveloped and enveloped viruses (with lipids and glycoproteins involved)^[^ ^36^ ^]^ the latter being the case for CoVs. Computational studies focusing on specific biological processes or interactions, such as the recector‐spike binding ACE2‐S‐RBD, require taking into account all these molecular details. For instance, molecular dynamics (MD) simulations on the ACE‐S‐RBD complex close to silane surfaces proved that the orientation of the complex respect the surface leads to variations in ACE‐S‐RBD binding energy (of ≈20*k*_*B*_ *T*).^[^ ^37^ ^]^ The adhesion of the full spike structure in human skin and hard surfaces^[^ ^38^ ^]^ have been studied using costly MD simulations, showing that the adaptability of the spike deformation is essential for its adhesion (particularly in soft substrates). Although these studies are quite informative, MD can only sample a small portion of the virus (the spike or quite often only portions of it) over short times. In this sense, the free energies estimated by MD mostly inform on the “enthalpy” of local and individual configurations, not sampling the large entropic contribution of the whole virus, via the coupled spike and envelope degrees of freedom. Modelling whole virus adsorption on fomites requires dedicated coarse graining models with tunable spike‐surface and membrane‐surface free energies and, quite importantly, a good representation of the spike and membrane configurational mobilities (coupled via the membrane fluidity and flexibility). As we show in this work, these features are essential to capture the large entropic contributions to the adsorption free energy, hidden in the spike and membrane internal motions.

In this work, we deploy AFM to study the adhesion of TGEV coronavirus on different reference surfaces by measuring the topographies of absorbed viruses,^[^ ^39^ ^]^ contact angles and virus surface coverage (trapping). The combination of these data with the virus concentration in solution, estimated via spectrophotometric evaluation of the genome, leads to Langmuir isotherms and the dissociation constants *K*_*d*_ for each surface. Joining experimental results with elastic theory and a coronavirus coarse‐grained model equipped with the relevant features mentioned above, we estimate the surface tension Σ, bending rigidity of the virus envelope κ, and the interfacial energy per area *W* associated to the virus membrane and the spikes. We conclude that the large affinity of the coronavirus to quite different surfaces is due to the seemly complementary physicochemical nature of the lipid envelope and the spikes.

## 2 Results

We studied the adhesion of TGEV on several reference surfaces including monocomponentsubstrates: mica, mica pretreated with poly‐L‐lysine (“Mica + PLL”), highly oriented pyrolytic graphite (HOPG), isopropanol‐cleaned silicon oxide (SiO_2_‐isopropanol), and plasma‐cleaned silicon oxide (SiO_2_‐plasma), and a bicomponent surface composed of SiO_2_ and Molybdenum disulfide, MoS_2_. These surfaces offer a relatively broad range of physico‐chemical interactions. Mica is negatively charged, so we expect to present electrostatic repulsion with the lipidic membrane, but also some attraction to the positively charged end‐domain of the spikes. By contrast, the treatment on mica‐PLL leads to a high positive charge (which would in principle enhance the interaction with negatively‐charged phosphate groups from lipids and attract the glycoproteins covering the spike sides). SiO_2_‐plasma, is hydrophilic with natural tendency to form hydrogen bonds, so it should present strong attraction for the membrane, as already observed in liposome adhesion studies.^[^ ^40^ ^]^ By contrast, SiO_2_‐isopropanol is mainly hydrophobic and neutral so both membrane or the spike might contribute with generally present (smaller) contributions from attractive dispersion forces. In the same way, HOPG is also neutral and very hydrophobic which might attract hydrophobic patches in the rather complex virus envelope. Finally, MoS_2_ is neutral and hydrophobic, but MoS_2_ is well known for having a strong affinity to the coronavirus spike^[^ ^41^ ^]^ due to the formation of disulfide bonds with cysteines in the spike.^[^ ^42^ ^]^

To assess the relevance of our findings on the TGEV, we carried out a comparison with a reference enveloped virus without spikes. In particular, we also investigate the adhesion of Haloarcula California icosahedral virus 1 (HCIV‐1) internal vesicle^[^ ^43^ ^]^ to mica, mica‐PLL, graphene and HOPG. The HCIV‐1 particle presents a radius of *R* = 33nm,^[^ ^43^ ^]^ which is not very far from that of the TGEV. Another similarity, which is important for a fair comparison on adhesion, is that both TGEV and the internal vesicle of HCIV‐1 are rather soft, with similar effective spring‐constant under AFM compression of ≈0.01 Nm^−1^.

### 2.1 Analysis of AFM Topographies

**Figure 1:**
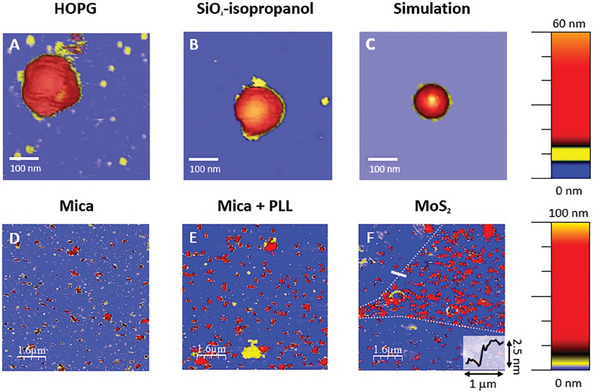
Examples of AFM topographical images of TGEV on various substrates. Panels A (HOPG) and B (SiO_2_‐isopropanol) illustrate single virus partiles highlighting their nearly spherical shape due to lipid envelopes. The image size is ≈500 × 500 nm^2^. Viruses still may differ in size due to pleomorphism and present different degrees of attachment to the surfaces, which will be the main focus of our analysis later. Panels A and B correspond to real virus with radii 53 and 50 nm, respectively. Panel C shows the virtual AFM image of a coarse‐grained virus model (with average radius *R*_*v*_ ≈ 43 nm). In panel C the structural details in yellow are the adsorbed viral spikes. Panels D (mica), E (mica + PLL) and F (MoS_2_) exhibit images with a 1/30 viral dilution on each surface (see also Supporting Information). AFM images for the same dilution of viruses attached to the other surfaces under study are displayed in Figure S1 (Supporting Information). Panel F includes a dotted white line to highlight MoS_2_ ‐ SiO_2_ boundaries, which can be easily seen by optical microscopy Figure S4 (Supporting Information), and the inlet caption shows a cross section of the solid white line, displaying the ≈2.5 nm thickness of the MoS_2_ flake.

AFM offers relevant information about the surface viral capture, as well as the size and shape of adsorbed viruses. **Figure** **1**A–C presents high resolution images of individual virus adsorbed at several surfaces (pixel size ≈5 nm). Larger AFM images (8 × 8 µm^2^) using low spatial resolution (pixel size ≈30 nm) as those in Figure 1D–F and Figure S1 (Supporting Information), were used to quantify viral capture. In the process of virus counting (see Experimental Section), we consider the particles which heights between 30 and 100 nm to avoid small portions of damaged viruses and large aggregates.

#### 2.1.1 Virus Size, Shape, and Deformation

**Figure 2:**
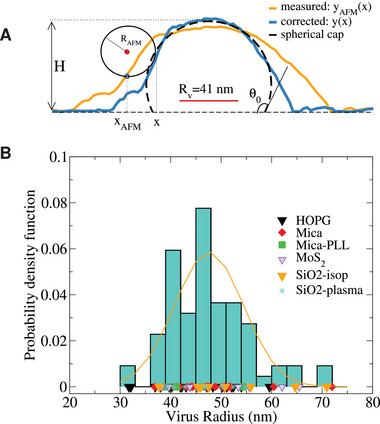
A) Measured AFM profile (orange line) and corrected profile taking into account AFM tip dilation and compression. The AFM tip is illustrated as a sphere of ≈25 nm. Results correspond to one TGEV particle on mica. The contour of the corrected profile *y*(*x*) is used in Equation (1) to provide the bare radius of the individual virus particle (*R*_*v*_ = 41 nm in this case, indicated with a red line). The black dashed line fits the corrected profile to a spherical cap, from which the contact angle θ_0_ is obtained. B) Probability density function of virus radii obtained from all the analyzed samples (colored symbols below indicate the lack of bias with different surfaces). The orange line indicates a Gaussian fit with average 47 nm and standard deviation 7 nm.

The average geometry of virus particles are necessarily convoluted with the relatively broad virus size polydispersity^[^ ^20^ ^]^ and tip dilation. ≈20 high‐resolution images of individual particles per surface (Figure 1) were used to draw 1D projection of the topography profiles *y*_*AFM*_(*x*) against the surface coordinate *x* (**Figure** **2**A). We correct the shape of the AFM profiles against tip dilaton in *x* direction and AFM compression in *y*, using the re‐scaling *y*(*x*) = λ_*y*_ *y*_*AFM*_(*x*/λ_*x*_) with λ_*x*_ = 1.4 and λ_*y*_ = 1.1 (see Experimental Section). Figure 2A illustrates the measured *y*_*AFM*_(*x*) (orange) and the corrected *y*(*x*) (blue) for the AFM topography of one TGEV particle over mica.

To evaluate the true radius of the spherical‐shaped virus, we use a volume‐based estimation which minimizes the assumptions on the shape other than the circular symmetry in the plane. The adsorbed particle volume is taken as half the volume of revolution associated with the profile *y*(*x*). The radius *R*_*v*_ associated to the “volume‐based” estimation is,

(1) $$\frac{4\pi }{3}R_{v}^{3}=V=\frac{1}{2}\int_{x_{0}}^{x_{1}}\pi y{\left(x\right)}^{2}dx$$

where the integration limits *x*_0_ and *x*_1_ are determined by the selected cutoff height *y*(*x*_0_) = *y*(*x*_1_) = *y*_*cut*_ (we used a reasonably small value *y*_*cut*_ ∈ (5 − 10) nm leading to small variations in *R*_*v*_). This approach is validated by using two virtual spherical AFM tip of 15 and 30 nm on a CG virus simulation (Experimental Section). This analysis resulted in *R*_*v*_ ≈ (45 ± 2) nm which is consistent with the real radius of the CG model 41 nm. Afterward, we applied Equation (1) to the experimental HCIV‐1 profiles to obtain, *R*_*v*_ = (29 ± 2) nm which is consistent with the size measured from chromatography (33 ± 2) nm.^[^ ^44^ ^]^

**Figure 3:**
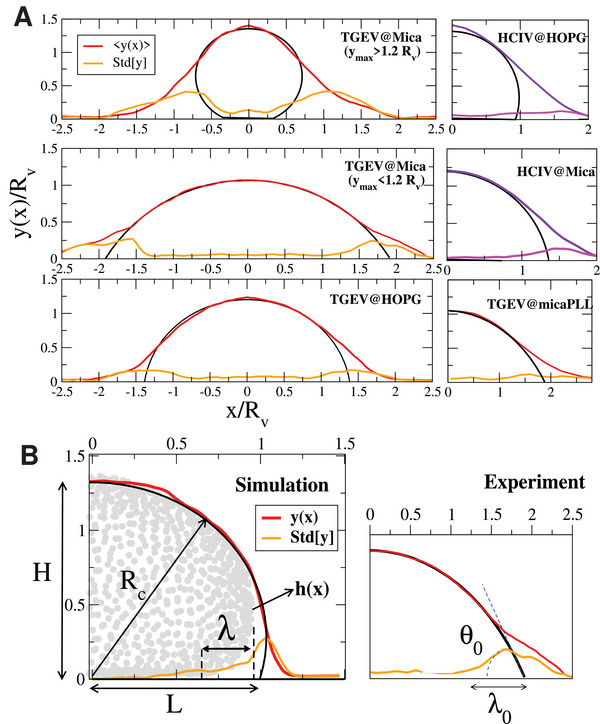
A) Average profiles and standard deviation Std[*y*] for TGEV (left) and HCIV‐1 vesicles (right) obtained from a collection of ≈20 AFM images. We illustrate the heterogeneity of TGEV profiles in mica, showing conditional averages for deflated particles with *y*_max_ < 1.2*R*_*v*_ and less deformed particles with *y*_max_ > 1.2*R*_*v*_. The spherical cap approximation is shown with black lines. B) The right panel shows a virtual‐AFM profile from the virus CG‐model (Experimental Section and Supporting Information). The circles indicate the location of the CG beads. The relation between the Std[*y*](*x*) profile and the extrapolation length $\lambda =\sqrt{\kappa /(2W)}$ is indicated. The right panel shows an experimental AFM profile, illustrating the determination of λ_0_ and contact angle θ_0_ from the spherical cap approximation (black line).

Once the size estimation methods have been validated, we analyzed the experimental topographical profiles of TGEV. Taking data from all surfaces and considering δ_*y*_ ≈ 5 nm for compression correction, we obtain *R*_*v*_ = (46 ± 3) nm for the TGEV particle. This value is consistent with the envelope radius measured from cryo‐EM *R*_0_ ≈ 41 nm.^[^ ^14^ ^]^ The histogram of the coronavirus radius, presented in Figure 2B, show a maximum range between 30 and 70 nm and standard deviation of 7 nm. The average and the radius range are consistent to those reported in Ref. [20] via cyro‐EM. As an aside, we also tested the more frequently used “surface‐based” estimation for the radius, *R*_*s*_ = (1/2) (*H* ^2^ + 2*L* ^2^)^1/2^, based on equating the bare surface $4\pi R_{s}^{2}$ to that of an adsorbed drop (spherical cap) with height *H* and width 2*L* extracted from the AFM profiles. This method consistently estimates the radius *R*_*s*_ ≃ *R*_*v*_ when it is applied to a CG vesicle model and to the HCIV‐1 vesicle (*R*_*s*_ = (31 ± 2) nm, Figure S2 (Supporting Information). Interestingly, *R*_*s*_ surpasses the TGEV particle size in ≈10 nm, due to the overestimation of the basal width 2*L*. Figure S2 (Supporting Information) indicates that this overestimation‐value Δ*L* is correlated to the presence of spikes adsorbed to each type of substrate. Thus, we use *R*_*v*_ in Equation 1 to estimate the individual radii of virus particles. Data for *R*_*v*_ permits to obtain the scaled virus height, *H*/*R*_*v*_ which will be a central quantity in the adhesion analysis. The averages 〈*H*/*R*_*v*_〉 were found to be 1.08 on mica‐PLL, 1.09 on SiO_2_‐plasma, 1.17 on SiO_2_‐isop, 1.20 on HOPG, 1.24 on MoS_2_ and 1.25 on mica, with error bars smaller than 0.03. The polydispersity in *H*/*R*_*v*_ (standard deviation over average) is small (≈0.05 on SiO_2_‐plasma) except for mica and MoS_2_ (≈1.4). **Figure** **3**A illustrates the relatively broad profile distribution observed in mica: from flattened *H*/*R*_*v*_ < 1.2 to slightly deformed particles, with *H*/*R*_*v*_ reaching up to 1.6.

#### 2.1.2 Adsorption Energy and Mechanical Properties from AFM Profiles

For a statistical analysis of the absorbed virus shapes, each AFM profile was normalized with its own radius (evaluated by Equation (1)) defining $\hat{y}=y/R_{v}$ and $\hat{x}=x/R_{v}$. Each profile was carefully centered at its maximum so as to evaluate the average, $\bar{\hat{y}}\left(\hat{x}\right)$, and the local standard deviation, $\mathrm{Std}\left[\hat{y}\right]\left(\hat{x}\right)$, for the collection of profiles on each surface.

Examples of AFM scaled averaged profiles for mica and HOPG are illustrated with red lines in Figure 3A. Despite being seldom reported, the local standard deviation (over AFM profiles), shown in Figure 3A, provides relevant information, as we explain below. Black lines in Figure 3 indicate the best fit to the averaged profiles using the spherical‐cap shape;^[^ ^45^, ^46^ ^]^ i.e. the arc *x* = *R*_*c*_cos (ϕ) and *y* − *y*_0_ = *R*_*c*_sin (ϕ), with *y*_0_ = *H* − *R*_*c*_ and *R*_*c*_ being the fitted curvature radius. The spherical cap fitting provides a first order estimation to the contact angle $\theta_{0}=\arccos\left(1-H/R_{c}\right)$,^[^ ^47^ ^]^ which corresponds to the drop‐like adsorption limit. The complete set of profiles is presented in Figure S3 (Supporting Information). To get insight on how a realistic virus shape *h*(*x*) translates into the average AFM profile *y*(*x*) and its standard deviation (Std), we have processed the virtual‐AFM profiles *y*(*x*) from the profiles *h*(*x*) obtained from CG simulations (Experimental Section and Supporting Information), using the same protocol as in the experiments. As shown in Figure 3B, *h*(*x*) runs over the contour of the virus. The projection of the virus CG‐beads in the xz plane (Experimental Section) are indicated by filled circles. For the sake of clarity, we have removed the spikes in this image. Moving from *x* = 0 to positive *x*, one notices that Std[*y*(*x*)] suddenly increases once the tangent of *h*(*x*) drops vertically (due to the vesicle‐like shape). In these border domains, the paths followed by the AFM tip present larger variations precisely because |*dh*(*x*)/*dx*| becomes very large (and eventually infinite at the maximal width of the path, Figure 3B). Quite interestingly, from Figure 3B the location where Std[*y*(*x*)] is larger, closely matches the non‐adsorbed portion of the virus, and as suggested from CG simulations (Figure 3B) it even indicates the shape of the profile *h*(*x*) near the surface. Moreover, the central region where Std[*y*(*x*)] is smaller (often close to zero) reveals the adsorbed surface of the virus. This information will be later used to estimate the ratio between the bending and the adsorption energy. In the case of the TGEV, the domains with large Std[*y*] are much more pronounced than those found for the HCIV‐1 vesicle (Figure 3A). This feature is quite probably due to the effect of the TGEV spikes, being consistent with the analysis in the Figure S2 (Supporting Information). A general feature in the shape of adsorbed vesicles, compared with that of liquid drops, is the formation of a domain with larger curvature close to the surface (Figure 3B). At a distance $\lambda ∼\sqrt{\kappa /W}$ from the detachment point, the elastic energy due to the vesicle curvature (zero in the adsorbed region) balances the adsorption energy. Figure 3B indicates λ in the CG virus model. Using the spherical cap shape as first‐order estimation, an analytical relation for the so‐called extrapolation length λ was derived in Ref. [47], $\lambda_{0}=\sqrt{2\kappa /W}\cot\left(\theta_{0}/2\right)$. Here λ_0_ is the distance from the point where the vesicle detaches from the surface to the intersection between the spherical cap and the substrate (Figure 3B, right). We use the location where Std[*y*(*x*)] increases to estimate the detachment point. The distance to the intersection of the fitted spherical‐cap with the surface (*z* = 0) provides λ_0_ (the distance to the maximum of Std[*y*(*x*)] could be another option, leading to very similar estimations). As an example, for HOPG in Figure 3B, we get θ_0_ ≈ 83° and λ_0_ ≈ 0.55*R*_*v*_ or 24 nm therefore, κ/*W* ∼ 270 nm^2^. Note that vesicles with radius smaller than (2κ/*W*)^1/2^ ≈ 23 nm prefer to be free than to be bound to the surface:^[^ ^5^, ^48^ ^]^ consistently, this lower cutoff radius (half the size of the average virus) is outside the range of the histogram of Figure 2.

Having the ratio κ/*W*, we need a sound estimation for either κ or *W*. To the best of our knowledge, the bending rigidity of the coronavirus envelope has not been so far estimated, so we start by arguing by analogy with similar vesicles. We can estimate κ using a theoretical relation between *W* and the flattening ratio *L*/*H* for strong adhesion (pancake regime), developed by Tordeux et al.^[^ ^47^ ^]^ and experimentally tested by Boxer et al.,^[^ ^49^ ^]^ with *R* = 50 nm phosphatidicholine liposomes. For large flattening ratios *L*/*H* ∼ 3, Boxer et al.,^[^ ^49^ ^]^ report *W* ∼ 1.5 × 10^−4^ Jm^−2^ on a glass substrate, i.e. essentially SiO_2_, and also predict *W* ∼ 5 × 10^−4^ Jm^−2^ for mica (upon Reviakine and Brisson results^[^ ^50^ ^]^). Combining our previous estimation κ/*W* ∼ 270 nm^2^ with *W* ∼ 1.5 × 10^−4^ Jm^−2^ we get κ ∼ 10 *k*_*B*_ *T*. The effective contact angles θ_0_ provide, via the Young–Dupré expression *W* = (1 + cos θ_0_)Σ, a relation between the surface adhesion *W* and the surface tension Σ of the virus envelope (**Table** **1**). For HOPG we get, Σ ≃ *W*/1.12 ∼ 1.33 × 10^−4^ Jm^−2^. Taking this value as reference and using *W* = (1 + cos θ_0_)Σ, we forecast the values of the adhesion parameter $WR_{v}^{2}/\kappa$ shown in Table 1. The estimated ranges *W* ∼ Σ ∼ 10^−4^ Jm^−2^ are consistent with the expected limits: first, Σ is smaller than the upper limit of lysis tension for the rupture of a bound vesicle (≈[10 − 50] × 10^−4^ Jm^−2^).^[^ ^48^ ^]^ And second, Table 1 shows that the values of the adhesion parameter are larger than the free vesicle threshold,^[^ ^5^ ^]^ below which vesicles prefer to be unbounded, i.e. $WR_{v}^{2}/\kappa >2$.

**Table 1:** Results for TGEV particles for the effective contact angle, ratio between the surface adhesion energy *W* and the virus envelope surface tension Σ and estimated adhesion parameter $WR_{v}^{2}/\kappa$ (see text). Results for the HCIV‐1 vesicles are shown in Table S1 (Supporting Information).

Surface	θ_0_	*W*/Σ = 1 + cos [θ]	$WR_{v}^{2}/\kappa$
Mica	96°	0.90	4.7
MoS_2_	88°	1.04	5.5
HOPG	83°	1.12	6.0
SiO_2_‐isop	78°	1.18	6.4
MicaPLL	57°	1.55	8.2
SiO_2_‐plasma	55°	1.56	8.3

It will be later shown these estimations for $WR_{v}^{2}/\kappa$ are in good agreement with a continuum elastic theoretical model derived from CG‐virus simulations. To conclude, note that the ordering of *W*/Σ values in Table 1 shows that the strongest adhesion energies correspond to SiO_2_‐plasma and mica‐PLL, being about twice larger than the weakest surface energies, MoS_2_ and mica.

Results for the scaled height, contact angle and adhesion work estimations of HCIV‐1 vesicles in HOPG, mica‐PLL, mica and graphene are shown in the Supporting Information. The HCIV‐1 envelope presents the smallest deformation on the hydrophobic and neutral HOPG (contact angle 105° ± 6 corresponding to *WR* ^2^/κ = 4.0 ± 0.2) while the strong electrostatic attraction in positively charged mica‐POLY to the negatively charged HCIV‐1 envelope induces the largest deformation (contact angle 65° ± 5 with *WR* ^2^/κ = 6.5 ± 0.5).

### 2.2 Viral Adhesion and Dissociation Constants

In this section, we present Langmuir adsorption isotherms to study the affinity of the TGEV particles to the substrates. One might expect that the dissociation constant *K*_*d*_ will correlate with the surface energy *W*, as it happens in vesicles.^[^ ^5^ ^]^ We advance that, surprisingly, this is not the case for the TGEV due to the significant complementary contribution of the spike adhesion.

#### 2.2.1 Monocomponent Surfaces

Adsorption isotherms of TGEV on different monocomponent surfaces were obtained by studying virus trapping on every substrate under increasing bulk concentration. The adsorption isotherm requires counting the virus particles trapped on the surface per square micron (virus capture, VC) from the free viruses in bulk, at different concentrations (Experimental Section and Supporting Information). Measurements were accomplished at a very low concentration, which avoids systematic errors such as the underestimation of VC due to virus aggregates.

**Figure 4:**
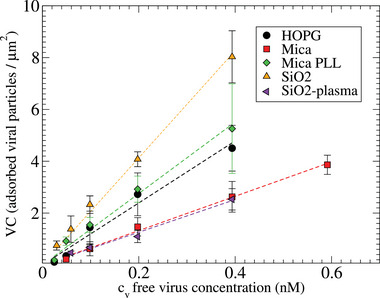
Hill–Langmuir adsorption isothermal showing the adsorbed viral particles per µm^2^ (VC) against the concentration of virus in bulk *c*_v_. Dashed lines are fits to the linear (Henry) regime. The error bars correspond to the standard error from data obtained from *n* = 6 images at each concentration.

Determining the molar virus concentration in solution is a non‐trivial task which has been solved by measuring the mass of genome in solution (Experimental Section). Each virus includes a single copy of the viral genome of mass 2.37 10^−17^ g, which permits us to calculate the number of viral particles in our sample. Viral concentrations ranged 3.56 × 10^11^ virus per mL (i.e. 1/10 dilution of original stock) to 1.48 × 10^10^ virus per mL (1/240 dilution). At these small concentrations, we found that the adsorption isotherms correspond to the Henry regime, where the number of captured particles is proportional to bulk concentration (**Figure** **4**). To estimate the dissociation constant *K*_*d*_ from the Langmuir model in the Henry regime we use Θ/Θ_max_ ≈ *c*_*v*_/*K*_*d*_, where Θ is the fraction of surface occupied by virus particles and Θ_*max*_ the maximum virus coverage. The fraction of virus‐occupied surface is defined as Θ = VC *A*_*v*_ where *A*_*v*_ is the area covered per virus, *A*_*v*_ = π*L* ^2^ (with *L* obtained from the spherical‐cap envelope, Figure 3). We consider Θ_*max*_ ≈ 0.64 which corresponds to the 2D random packing fraction. Hence, *K*_*d*_ = Θ_max_/(*a*π*L* ^2^), being *a* the slope of the curves in Figure 4. These slopes provide the virus capture ability for each surface, ordered as: SiO_2_‐plasma 6.3 Virus/(µm^2^nM); mica (6.6), HOPG (11.9), mica‐PLL (13.7) and SiO_2_‐isop (20.6). These numbers result in *K*_*d*_ ∼ 5nM which is ≈10 times the virus concentration in solution and within the Henry regime (**Table** **2**).

**Table 2:** Virus captured per micron square surface and nano‐molar virus concentration, percentage of total viruses captured onto the surface, the virus dissociation constants *K*_*d*_ and net adsorption free energy Δ*G* on different surfaces. MoS_2_ *K*_*d*_ values were obtained in bicomponent surfaces, from differential adsorption with respect the SiO_2_ background (which is similar in properties to isopropanol‐cleaned SiO_2_). In this case we extrapolate from VC the effective percentage of capture (in brackets). To estimate *K*_*d*_ we used an area per virus π*L* ^2^ given by the basal radius *L* of the spherical cap and Θ_max_ = 0.64 (random packing limit). The standard error in VC is ≈1 virus/(µm^2^nM) (similar in all surfaces) and corresponds to a sample with *n* = 30 images (six images for each ‐five‐ virus concentration on each surface. Error analysis for *K*_*d*_ and Δ*G* is presented in Experimental Section.

Surface	VC = Virus/([µm^2^ nM]	% of capture	*K*_*d*_ [nM]	Δ*G*/*k*_*B*_ *T*
MoS_2_	81	(25%)	1.2 nM	−20.5
SiO_2_‐isopropanol	21	6.30%	4.9 nM	−19.1
Mica + PLL	14	3.97%	4.0 nM	−19.3
HOPG	12	2.87%	9.8 nM	−18.4
Mica	7	1.75%	20.9 nM	−17.7
SiO_2_‐plasma	6	1.72%	8.2 nM	−18.6

#### 2.2.2 Bicomponent Surfaces MoS_2_ and SiO_2_

For the estimation of *K*_*d*_ on MoS_2_ surfaces we used a different approach based on differential adsorption. To that end we captured virus particles on bicomponent surfaces composed by MoS_2_ flakes (typically of 10µm^2^) on a SiO_2_ background, as illustrates Figure S4 (Supporting Information). MoS_2_ flakes are identifiable in AFM topographies as boundaries with an increase in height (Figure 1F, inset). Drops with controlled virus dilution were incubated on these bicomponent surfaces to study the differential virus adsorption. As shown in Figure 1F, we found ≈4 viruses attached to the MoS_2_ flakes for one virus deposited onto the SiO_2_ background. This ratio 4 : 1 was found to be similar for all the viral dilutions considered, which confirms that adsorption events in both patches are fully independent, without second‐order kinetics or competition effects. Furthermore, as shown in Figures S5 and S6 (Supporting Information), both the viral capture and the distribution of virus heights in the SiO_2_ background, are compatible with those measured in monocomponent SiO_2_‐isopropanol surfaces. This permits us to use the *K*_*d*_ obtained for pure SiO_2_‐isopropanol to estimate that of MoS_2_. As the virus concentration in bulk is the same above each patch of the bicomponent surface, the result for differential adsorption leads to *K*_*d*_(MoS_2_) ≈ (1/4)*K*_*d*_(SiO_2_isop).

#### 2.2.3 Dissociation Constant *K*_*d*_ and Adhesion Free Energy Δ*G*

With this last result at hand, we provide in Table 2 the estimated values of *K*_*d*_ for the different surfaces. Larger values of *K*_*d*_ correspond to smaller adsorption affinities. The free energy of adsorption Δ*G* at the standard concentration [1M], is usually presented as *K*_*d*_/[1*M*] = exp [βΔ*G*], where β = 1/*k*_*B*_ *T*. We find Δ*G* ∼ −19 *k*_*B*_ *T* with variations of ≈4 *k*_*B*_ *T* between surfaces; a range which is consistent with medium‐size vesicles.^[^ ^10^ ^]^ To put some examples based on Langmuir isotherms, Ref. [51] reported Δ*G* = −23.7 *k*_*B*_ *T* for unilamellar vesicles of average diameter of 100 nm on functionalized surfaces and Murray and Park^[^ ^22^ ^]^ report Δ*G* ≈ −22 *k*_*B*_ *T* for the adhesion of poliovirus (50 nm in diameter) on SiO_2_.

Yet, a surprise arises when comparing the results for *K*_*d*_ or Δ*G* in Table 2 with those obtained for the effective adsorption energy *W* in Table 1. There is no clear correlation between *W* and *K*_*d*_. In fact, the opposite trend tends to be the case, as the substrate with the smallest affinity for TGEV is the one presenting the largest effective adsorption energy (SiO_2_‐plasma, Δ*G* = −18.6 *k*_*B*_ *T* and $W=8.3\;\kappa /R_{v}^{2}$). And, vice versa, the MoS_2_ surface (Δ*G* = −20.5 *k*_*B*_ *T* and $W=5.5\;\kappa /R_{v}^{2}$) presents a large affinity for the TGEV, but a relatively large virus height upon adhesion, which is compatible with a small adhesion energy *W*.

### 2.3 Theory and Coarse‐Grained Simulations

**Figure 5:**
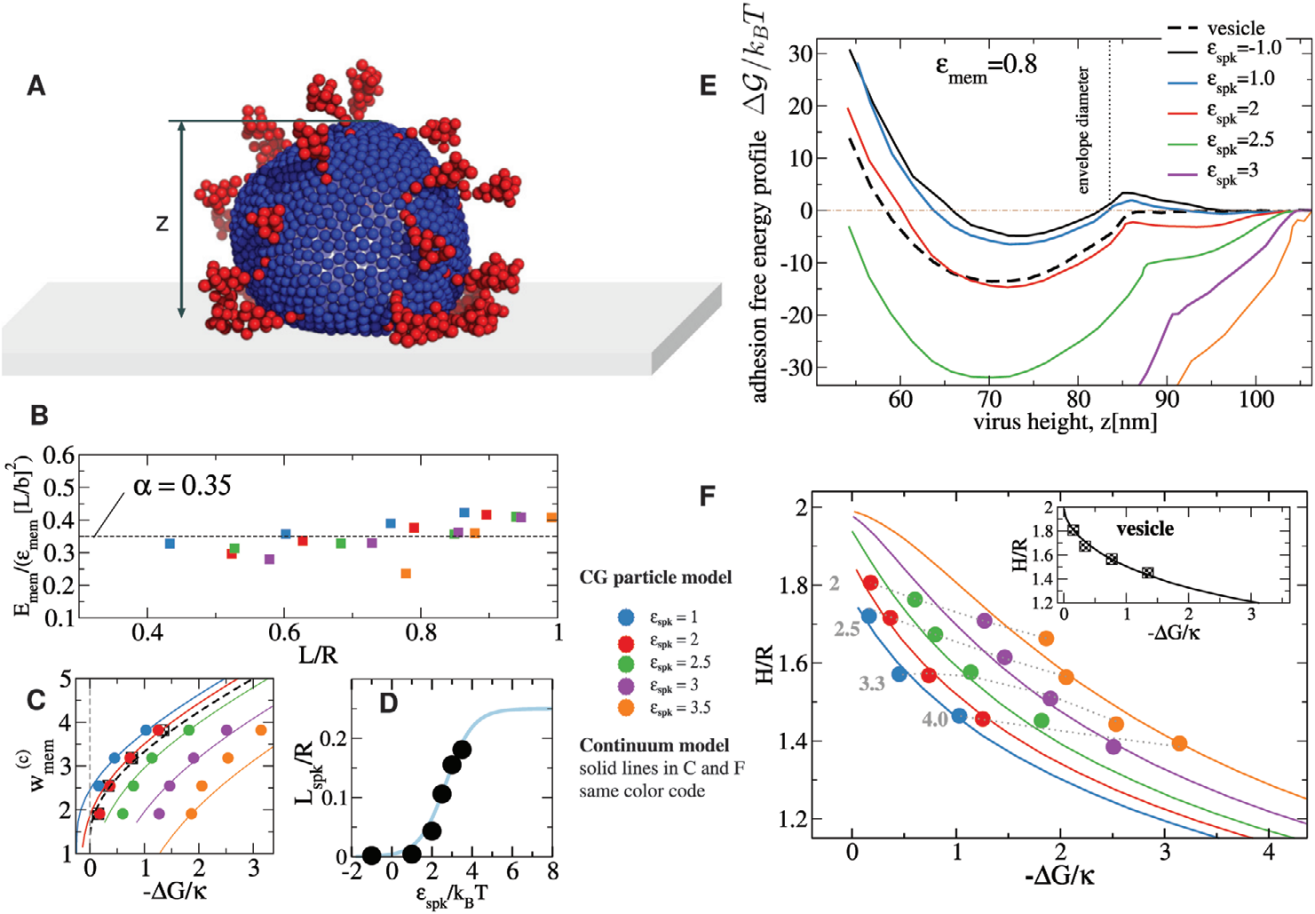
A) Illustration of the CG virus model. The membrane is composed by self‐aggregating mobile polar beads of radius *b* ≈ 2 nm and the spike is an elastic‐network with realistic values of flexibility and diffusivity (Experimental Section and Supporting Information). The adhesion energy per bead is noted as ε_mem_ (blue) for the envelope and ε_spk_ for the spikes (red) (the figure corresponds to ε_*spk*_ = 3.0 *k*_*B*_ *T* and ε_*mem*_ = 0.8 *k*_*B*_ *T*). B) Average membrane adhesion energy scaled with π*L* ^2^ε_mem_/(π*b* ^2^); the dashed line corresponds to the mean value α = 0.35. The color‐coded filled circles indicate the energy of the spike CG beads ε_spk_ (in *k*_*B*_ *T* units). C) Envelope non‐dimensional adhesion energy at contact *w* ^(*c*)^ = ε_mem_ *R* ^2^/(π*b* ^2^κ) against the total adsorption free energy. D) Effective spike adsorption length ℓ_spk_ ≡ *L*_spk_/*R* = *E*_spk_/[2ε_spk_ *LR*/*b* ^2^]; where *E*_spk_ is the total spike adhesion energy. The solid line is a fit to the adsorbed population of a Hill model ℓ_spk_ = ℓ_max_ *p*/(1 + *p*) with ℓ_max_ = 0.25 and $p=e^{-n\;\left(\left[-\varepsilon_{\mathrm{spk}}\right]-\mu_{0}\right)/k_{B}T}$ with Hill index *n* = 1.2 and chemical potential for unbounded spikes μ_0_ = −3.3  *k*_*B*_ *T*. E) Free energy profiles ΔG(z) associated to the envelope maximum height *z* (see A), obtained from umbrella sampling (ε_mem_ = 0.8*k*_*B*_ *T*). F) The CG virus height *H*/*R* against the unbounded‐bounded net free energy difference −Δ*G*/κ scaled with the CG‐model bending rigidity κ ≈ 40*k*_*B*_ *T* (Supporting Information). The inset corresponds to the CG‐vesicle (no spikes). Lines in (B) and (E) corresponds to the theoretical model in Equation (2). In (F) the gray figures (and dotted lines) indicate the isolines of $w_{\text{mem}}^{\left(c\right)}=R^{2}W_{\mathrm{mem}}^{\left(c\right)}/\kappa$.

We now present results from a fairly realistic particle‐based coarse‐grained (CG) model of the coronavirus and an analytical approach based on a continuum model based on a Helfrich‐type free energy, generalized to include spikes. Analysis of CG simulations permit us to extract essential insights on the viral adhesion. We insert such information in the continuum model so that it provides quantitative agreement with particle simulations (**Figure** **5**C,F). By combining both approaches, we are able to match experimental results for the CoV and extract the adhesion energies of the membrane and spikes on different surfaces.

#### 2.3.1 Coarse‐Graining Simulations of Adsorbed TGEV Coronavirus

Coarse graining models have been extremely useful to understand the spike structure and fluctuations^[^ ^52^ ^]^ and also the virus diffusive dynamics and its role in infection.^[^ ^53^ ^]^ We performed an analysis of the TGEV adsorption using a CG model illustrated in Figure 5A (Experimental Section). Details and validations of the different components of the CG virus model (envelope, spikes and surface) are presented in the Figures S8 and S9 (Supporting Information). Our CG is composed of beads with effective radius *b* ≈ 2 nm, used to build the lipid envelope and the spikes. The lipid envelope is modeled using a single layer of beads interacting with nearest neighbor dipolar potentials and lateral interactions based on central forces. A relevant novelty of this model, essential for this work, is to capture the fluidity, flexibility and bending modulus of the lipid membrane.^[^ ^54^, ^55^ ^]^ Importantly, the model allows the spikes to diffuse over the envelope^[^ ^56^ ^]^ (Video S1, Supporting Information). To this end, each spike is bonded to one CG‐lipid of the fluidic membrane (bead #4 in Figure S8, Supporting Information), which can therefore freely diffuse. The spike structure is adapted from previous models^[^ ^52^, ^57^ ^]^ and interactions are tuned to reproduce the flexibility observed in cryo‐electron tomograms^[^ ^58^, ^59^ ^]^ (Supporting Information). A critical aspect of our model is the interaction of membrane and spike beads with the substrate, modeled by a potential well of depth ε per CG‐bead. Specifically, the interaction energy values for the lipid membrane beads were chosen to be ε_mem_/*k*_*B*_ *T* = {0.6, 0.8, 1.0, 1.2} and those for the spike beads were ε_spk_/*k*_*B*_ *T* = {− 1.0, 1.0, 2.0, 2.5, 3.0, 3.5}. It is important to note that positive values of ε correspond to attractive bead‐substrate interactions, which are obviously required for adsorption. The negative value of ε_spk_ indicate a purely repulsive interaction of the spike with the surface (Experimental Section and Supporting Information). Our model allows tuning the bending rigidity κ of the virus envelope, whose value is connected with the number of beads used for the CG vesicle, i.e., with the membrane area‐per‐bead, *A*_*bead*_. An effective bead radius *b* ≈ 2 nm (Supporting Information) leads to *A*_*bead*_ = π*b* ^2^ ≈ 12.50 nm^2^ which limits the lowest value of the membrane bending rigidity to κ ≃ 40 *k*_*B*_ *T* (Figure S9, Supporting Information). Reaching smaller values (κ ∼ 10 *k*_*B*_ *T*) require smaller beads, which prohibitively increases the cost in simulation time. As the model does not consider molecular details below ≈4 nm, it does not resolve lipid protrusion fluctuations (one CG bead corresponds to ≈ 30 membrane lipids, Experimental Section and Supporting Information). Heterogeneity in hydrophobicity, partial charge along the spike or lipid envelope and solvent (water) degrees of freedom are neither resolved. Thus, a great deal of solvent, spike and lipid‐related entropy are encoded inside the effective interaction energies ε. Despite these limitations, the CG model offers essential features to understand the different roles of the envelope and the spikes in the adhesion of CoV on surfaces.

We start by analyzing the relation between the adhesion energy and the shape of a virus with nominal radius *R*, determined by its height *H* and radius of contact on the surface *L* (Figure 3B). A reasonable ansatz for the membrane adhesion energy is *E*_mem_ = π*L* ^2^[αε_mem_/(π*b* ^2^)], where we use the spherical cap approximation for the contact area *A*_*c*_ ≈ π*L* ^2^. Note that ε_mem_ is the minima of the bead‐surface potential (Supporting Information), which corresponds to the adhesion energy density at contact $W_{\text{mem}}^{\left(c\right)}=\varepsilon_{\text{mem}}/\left(\pi b^{2}\right)$. As pointed out by Lipowski,^[^ ^60^ ^]^ due to thermal fluctuations, the average energy in microscopic particle‐based CG descriptions of the surface‐membrane interaction (Supporting Information) is a factor α < 1 smaller than the contact value used in continuum descriptions. Our ansatz is verified in Figure 5B, by plotting the equilibrium average of *E*_mem_ scaled with ε_mem_(*L*/*b*)^2^ against *L*/*R* for different spike and membrane interaction energies. We find that α ≈ 0.35, which it is indeed roughly constant, with a small increasing trend with *L*. Thus, the relation between the non‐dimensional average adhesion energy and the energy per CG blob is *w*_*a*_ = α *w* ^(*c*)^ = α ε_*a*_ *R* ^2^/(π*b* ^2^κ) ≈ 1.11 ε_*a*_, where *a* = spk (spike) or mem (membrane). Values at contact, $w_{\text{mem}}^{\left(c\right)}$ are shown in Figure 5C against the net free energy Δ*G*, presented below.

The net spike adhesion energy is considered to scale as *E*_spk_ = 2π*LL*_spk_ε_spk_/(π*b* ^2^). This relation assumes that the spike‐surface contact area is proportional to the adsorbed perimeter 2π*L* times an effective length, *L*_spk_ which may depend on ε_spk_. This dependence is clearly observed in Figure 5D where ℓ_spk_ ≡ *L*_spk_/*R* = [*E*_spk_/ε_spk_][*b* ^2^/(2*LR*)] is plotted against ε_spk_/*k*_*B*_ *T*. The solid line is a fit based on a Hill adsorption isotherm model: $\ell_{\text{spk}}=\ell_{\text{spk}}^{\max}p/\left(1+p\right)$, with *p* = exp [−*n* Δε/*k*_*B*_ *T*], where Δε = (− ε_spk_) − μ_0_ is the energy difference between the bounded and the unbounded spike, with chemical potential μ_0_.^[^ ^61^ ^]^ The fit in Figure 5D yields *n* = 1.2. Since the spike‐spike interaction is not attractive the small degree of cooperativity is probably due to a small increase in the contact perimeter as ε_spk_ increases. We also find $\ell_{\text{spk}}^{\max}\approx 0.25\;R$ and μ_0_ ≈ −3.3*k*_*B*_ *T*, which are consistent with the spike length and excluded area (i.e., μ_0_ ∼ −*k*_*B*_ *T* ln [*A*_*cap*_/*A*_spike_] with *A*_*cap*_ = 2π *R*_*c*_ *H* and *A*_*spike*_ ∼ π(10nm)^2^).

We next use umbrella sampling (Experimental Section),^[^ ^62^ ^]^ to measure the profile of free energy difference between the bounded and unbounded state ΔG(z) of CoV CG model (Figure 5E) as a function of the maximum height *z* of the lipid envelope (Figure 5A). Solid lines correspond to adhesion‐free energy profile ΔG(z) for the CG virus model, and the dashed line correspond to the CG vesicle (i.e. the membrane envelope without spikes). The spikes introduce several relevant effects. The free energy well becomes more negative (attractive) as the spike adhesion energy is increased ε_spk_ > 0. However, the spikes also introduce a substantial effective repulsion of purely entropic origin. This steric interaction with the surface increases in ≈10 *k*_*B*_ *T* the binding free energy with respect to a vesicle (this value probably increases with the number of spikes). Notably, as shown in Figure 5E, even virus with mildly attractive spikes (ε_spk_ = *k*_*B*_ *T*, blue) present ≈10 *k*_*B*_ *T* less adhesion free energy than a naked vesicle (dashed line). The free energy of a naked envelope is in fact similar to that of a virus with rather sticky spikes ε_*spk*_ = 2.0 *k*_*B*_ *T*. For ε_*spk*_ < 2 *k*_*B*_ *T*, we find a potential barrier around *z* ∼ 2*R*_*v*_ ∼ 85nm, whose value reaches *E*_*b*_ ∼ 5 *k*_*B*_ *T*. Such a barrier should lead to a significant delay in the adhesion process, similar to a reaction‐limited process, with a prefactor $e^{E_{b}}$ multiplying the diffusion‐limit characteristic time.

To connect the free energy profiles of Figure 5E with the dissociation constant *K*_*d*_, we evaluate the average adhesion free energy ΔG∝∫ΔG(z)exp[−βΔG(z)]dz, where the integral runs over the simulation box. The result is quite close to the minimum of the profile, $\Delta G\approx \Delta G_{\min}$. Results for Δ*G* from CG simulations are presented (filled circles, Figure 5C,F) along with the results of the continuum elastic model (solid lines) presented below (Equation (2)). A first important message of Figure 5F is that the particle height *H* strongly varies with the membrane adhesion energy (indicated by gray numbers in Figure 5F), but it does not greatly vary with the spike adhesion energy (colored coded circles, Figure 5F). Another relevant information concerns the critical adhesion energy density *w*_*cr*_ needed for a stable capture (Δ*G* > 0). For the CG vesicle, we find unbinding for $w_{\text{mem}}^{\left(c\right)}<w_{cr}$ where $w_{\text{mem}}^{\left(c\right)}\equiv R^{2}W_{\text{mem}}^{\left(c\right)}/\kappa$. For the CG model, the critical adhesion parameter is *w*_*cr*_ ≈ 2, which is in excellent agreement with theoretical predictions based on continuum elasticity.^[^ ^48^, ^60^ ^]^ This unbinding transition at finite adhesion energy have energetic and entropic origins. For *w* ^(*c*)^ < *w*_*cr*_, the elastic energy penalty created at the vesicle‐surface contact line, with curvature λ^−1^ = [2*W*/κ]^1/2^, overpowers the adhesion energy. Close to the transition (*w* ^(*c*)^ ⩾ *w*_*cr*_) the contact area roughly scales as *A*_*c*_/*R* ^2^ ∼ Δ*w* with Δ*w* = *w* − *w*_*cr*_. Similar to the Casimir effect,^[^ ^10^ ^]^ a positive free energy density associated to membrane fluctuations *w*_*fluc*_ ∼ (*k*_*B*_ *TR*/κ*d*)^2^^[^ ^63^ ^]^ is created due to the reduction of the membrane undulations entropy, when confined in a layer of width *d*.^[^ ^60^ ^]^ Energetic unbinding yields *w*_*cr*_ = 2 and dominates for small particles (below 100 nm size).^[^ ^60^ ^]^ Yet, for the CG vesicle we get *w*_*fluc*_ ∼ 1 for an adsorbed layer of *d* ∼ 1nm, which is not negligible. Interestingly, we observe that the presence of adsorbed spikes slightly increases *w*_*cr*_ (see comments on Eq. (2) below), which could be consistent with an increase of the local curvature at contact, or an increase in the steric undulation repulsion by reducing *d*.

#### 2.3.2 Continuum Elastic Theory for CoV Adsorption

We developed a simple continuum model for coronavirus adsorption which permit us to connect with the experimental results and the CG virus model. The virus envelope is approximated as an elastic spherical cap with base radius *L*, height *H* and curvature radius *R*_*c*_ = *H*/2 + *L* ^2^/2*H*. The envelope area is taken to be constant over the adsorption process (an approximation commonly used for lipid vesicles^[^ ^46^, ^48^ ^]^): hence 4π*R* ^2^ = π*L* ^2^ + *A*_*cap*_ with *A*_*cap*_ = 2π*R*_*c*_ *H* the spherical cap area. This yields, *L* = (2 − *H* ^2^/2)^1/2^ which determines the envelope adhesion energy −*W*_mem_π*L* ^2^. The spike‐surface interaction energy −*W*_spk_π*LL*_spk_ is proportional to the perimeter of the contact line 2π*L* and the surface‐projected effective spike length, *L*_spk_ discussed in Figure 5D. The elastic energy is proportional to the bending rigidity κ, and it follows from the Helfrich–Canham description.^[^ ^60^, ^63^ ^]^ In describing the elastic energy, the spherical cap approximation becomes valid in the pancake regime (highly deformed particle) observed for the virus particles. Therefore we opt for a simplification of the theoretical model, which does not explicitly describe the elastic energy excess close to the contact line with curvature (2*W*/κ)^1/2^.^[^ ^60^ ^]^ Instead, we take into account elastic and entropic repulsive effects (due to the reduction of the membrane undulation entropy) by introducing an effective “unbinding” free energy density *w*_*cr*_ which induces a shift in the membrane adhesion energy as Δ*w* = *w*_mem_ − *w*_*cr*_. As shown in Figure 5F, this approximation (colored lines) quantitatively matches the results from CG simulations (filled circles), even close to the unbinding transition (Δ*G* > 0 in Figure 5C). The work of compression is −Δ*P* [4π*R* ^3^/3 − *V*_*cap*_], with deformed envelope volume *V*_*cap*_ = (π*H* ^2^/3)(3*R*_*c*_ − *H*) and internal excess pressure Δ*P*. We use κ and *R* = *R*_*v*_ as reference units to obtain non‐dimensional quantities: *g* = *G*/κ, *h* = *H*/*R*, ℓ = *L*/*R*, *r*_*c*_ = *R*_*c*_/*R*, *a*_*cap*_ = *A*_*cap*_/*R* ^2^ and the volume change δ*v* = (*V*_*cap*_/*R* ^3^) − 4π/3 with κ_*p*_ = Δ*PR* ^3^/κ. Finally, *w*_a_ = *W*_a_ *R* ^2^/κ, with *a* either membrane or spike, and *W*_*a*_ the average adhesion energy density. The analysis of the CG virus model leads to the following the continuum version of the adsorption‐free energy,

(2) $$g=-\Delta w_{\text{mem}}\;\pi \ell^{2}+\frac{1}{2}a_{cap}{\left(2r_{c}^{-1}-c_{0}\right)}^{2}-\kappa_{p}\delta v+g_{\text{spk}}$$

where the spikes' contribution contain an adhesive and a repulsive depletion term: *g*_spk_ = −*w*_spk_ 2πℓℓ_spk_ + *g*_*steric*_. From Figure 5E we take the spikes steric interaction with the surface as *g*_*steric*_ = 10*k*_*B*_ *T*/κ and ℓ_spk_ = *L*_spk_/*R* is taken from Figure 5D. Also, the best fit to CG‐model results in Figure 5C yields *w*_*cr*_ = 2 + 0.1*w*_spk_.

**Figure 6:**
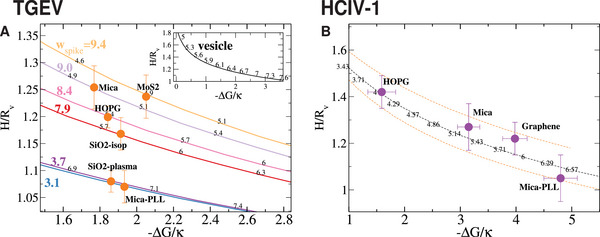
Scaled particle height against the scaled adsorption‐free energy (Δ*G*/κ < 0). A) Experimental results for Δ*G* for TGEV (circles) are scaled with κ = 10 *k*_*B*_ *T*. Lines correspond to the theoretical model in Equation (2) using *c*_0_ = 1 and κ_*p*_ = 3. Values of $W_{\text{mem}}^{\left(c\right)}R^{2}/\kappa$ are indicated with small black figures and $W_{\text{spk}}^{\left(c\right)}R^{2}/\kappa$ indicated with large colored figures. The inset shows the corresponding theoretical prediction for $W_{\text{spk}}^{\left(c\right)}=0$, i.e. no spikes (a vesicle), over the range of adhesion energy density predicted for the virus. B) Experimental results for HCIV‐1. Lines correspond to Equation (2) using *c*_0_ = 0.1 and κ_*p*_ = 6.5 (black) (results for κ_*p*_ = 6.0 and 7.5 are indicated with orange lines, respectively below and above). In B the values for −Δ*G*/κ are estimated from the scaled adhesion energy $W_{\text{mem}}^{\left(c\right)}R^{2}/\kappa$ measured from the contact angles obtained from the AFM indentation profiles. Error bars for *H*/*R*_*v*_ corresponds to the standard error of (A) samples of *n* = 20 viruses (except for mica‐PLL and SiO_2_‐plasma, *n* = 10) and (B) samples of *n* = 20 viruses.

**Figure 7:**
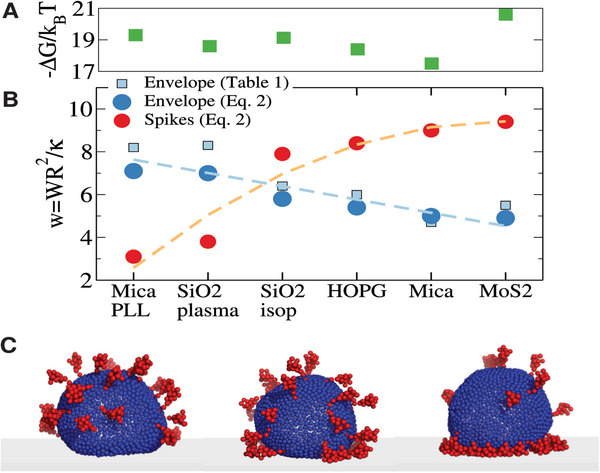
Illustration of the different conformations of the coronavirus according to the spike and membrane adhesion at each surface. A) Experimentally measured adhesion free energy −Δ*G*/*k*_*B*_ *T*. B) Circles indicate the values of $w_{\text{spk}}^{\left(c\right)}$ and $w_{\text{mem}}^{\left(c\right)}$ derived from Equation (2) which match the experimental values of *H* and Δ*G*, as shown in Figure 6. Squares correspond to $w_{\text{mem}}^{\left(c\right)}$ derived from the analysis of AFM profiles (Table 1). Dashed lines, drawn to guide the eye, highlight the opposite trends for $w_{\text{spk}}^{\left(c\right)}$ and $w_{\text{mem}}^{\left(c\right)}$. C) CG simulations for a case with large membrane adhesion (left), large spike adhesion (right) and similar adhesion (middle). The dynamics of these three cases are illustrated in Videos S1, S2, and S3 (Supporting Information).

In order to compare the theoretical model with the CG‐virus model, we use κ = 40*k*_*B*_ *T* and a small spontaneous curvature, *c*_0_ = 0.2, consistent with the vesicle model (Experimental Section and Supporting Information). In the CG model, the excess in internal pressure is solely due to surface tension: from the Laplace relation Δ*P* = 2Σ/*R* and thus κ_*p*_ = 2Σ*R* ^2^/κ. The lateral bead‐bead interaction energy is about *k*_*B*_ *T*, so Σ ≃ *k*_*B*_ *T*/(π*b* ^2^) ≈ 3.1 × 10^−4^Jm^−2^. This yields κ_*p*_ = 6.3 which leads to an excellent agreement with the CG‐results. Figure 5C,F shows quantitative agreement between the model in Equation (2) (solid lines) and the free energy Δ*G* from umbrella sampling simulations on the CG virus model. This gives us confidence to deploy the theoretical model for the analysis of the experimental results for the TGEV. The thick virus envelope, containing multiple types of membrane proteins (E and M) presents a large spontaneous curvature.^[^ ^64^ ^]^ Thus we choose *c*_0_ = 1 and the previously estimated κ = 10*k*_*B*_ *T* which leads to κ_*p*_ = 3 as the best fit to experimental results using Equation (2). It is important to remark that using these parameters in Equation (2), we reproduce the envelope adhesion energies estimated from AFM profiles in Table 1 (**Figures** **6** and **7**). The estimation for κ_*p*_ is consistent with the Laplace pressure induced by the above reported surface tensions, Σ ∼ 10^−4^Jm^−2^. Note that TGEV particles are fully filled with a protein‐condensed RNA chain, which barely creates osmotic pressure. The compression response of the TGEV particle under AFM indentation is thus far from the “soft” (κ ≈ 14*k*_*B*_ *T*) but pressurized lipid vesicles studied by Wuite's et al.,^[^ ^46^ ^]^ which reached MPa internal pressures due to a large concentration of impermeable osmolites (free lipids). In fact, under compression, the TGEV acts more like a soft polymer nanodroplet. AFM indentation curves reveal that the effective spring constant for TGEV compression is *k*_*sp*_ ≈ 0.01 Nm^−1^,^[^ ^14^ ^]^ similar to SARS‐CoV‐2 (0.013 Nm^−1^).^[^ ^16^ ^]^ This value is consistent with a soft polymeric material^[^ ^65^ ^]^ with Young modulus *E* ∼ *k*_*sp*_/*R* ∼ 200 kPa. In fact, we verified that for this Young modulus range, the elastic theory for soft nanodroplets^[^ ^65^ ^]^ reproduces the experimental values of *H*, Δ*G* shown with circles in Figure 6. An exploration of this dual characterization (vesicle‐like versus nanopolymer‐like) is left for future work.

The analysis of the adhesion energy densities is particularly interesting. Results of the continuum model of Equation (2) are presented in Figure 6 (solid lines). Figure 7 collects results for Δ*G* and the non‐dimensional adhesion energies $w_{a}^{\left(c\right)}=W_{a}^{\left(c\right)}R^{2}/\kappa$ for each surface and highlights the excellent agreement between Equation (2) and the values for $w_{\text{mem}}^{\left(c\right)}$ in Table 1, obtained from the analysis of AFM‐profiles (Figure 7B). In the case of the spikes, our estimations are in the range *W*_spk_ ∼ [1 − 3] × 10^−4^Jm^−2^; which is consistent with the Derjaguin approximation on the pulling force experiments of isolated spikes using AFM^[^ ^66^ ^]^: *W* = *F*/(2π*R*_*tip*_) ∈ [8 − 20] × 10^−4^ Jm^−2^ for silicon‐oxide tips and *W* < 3 × 10^−4^ Jm^−2^ for other metal oxides.

Figure 6 shows the interplay between the membrane and spike adhesion energies of TGEV in different surfaces. Membrane‐substrate energies (indicated by small black figures over the lines) range between $w_{\text{mem}}^{\left(c\right)}\approx 5$ for MoS_2_ and mica to $w_{\text{mem}}^{\left(c\right)}\approx 8$ for SiO_2_‐plasma and mica‐PLL where the virus is more deformed (lowest *H*). As shown in the inset of Figure 6 under such a range of adhesion energies, a standard vesicle would undergo a large modification in shape, from a small distortion *h* = *H*/*R* ≈ 1.8 to a pancake shape *h* ≈ 1.0 at $w_{\mathrm{mem}}^{\left(c\right)}\approx 8$. As shown in Figure 6B this typical trend for vesicle adhesion is also observed for the HCIV‐1 particle (internal vesicle), obtained from a virus with a lipid envelope, but without spikes. In this case we use Equation (2) with zero spike energy, and estimate Δ*G*/κ by placing the experimental values of the adhesion work *W* (measured from the contact angle obtained from AFM virus profiles) on the theoretical values (small figures over the black‐dashed line). As expected, for the HCIV‐1 internal vesicles, large deformations are related to large adhesion energies. However, the standard deformation‐adhesion relation is not observed in the TGEV, where the spikes make the difference: in fact, the spike adhesion energy is stronger $w_{\text{spk}}^{\left(c\right)}\approx 9.4$ in those surfaces with the smallest membrane adhesion (MoS_2_ and mica) while *w*_spk_ is smaller for surfaces with strong membrane‐substrate attraction (SiO_2_‐plasma and mica‐PLL). In the discussion section we argue that this adhesion “adaptability” of the TGEV to quite different surfaces is due to the different electrostatic nature of the spike and the membrane envelope.

### 2.4 Discussion

Using AFM, coarse‐grained simulations and a theoretical approach, this work evaluates the adhesion‐free energy and affinity of coronavirus (TGEV) to different substrates, as well as virus mechanical properties, such as bending rigidity, deformation and surface energy per area *W* (Table 1). Additionally, using AFM images for virus counting and a precise mapping of the virus bulk concentration via its genome mass, we draw Langmuir isotherms and measure dissociation constants *K*_*d*_ and adhesion‐free energies Δ*G* ∼ −20*k*_*B*_ *T* for different surfaces (Table 2). Our findings can be discussed in several lines. In the applied side, our results confirm that surface cleaning and functionalization can be an efficient way to modulate viral attachment, either toward a higher degree of viral capture or toward the prevention of viral adsorption. Although seemly small, the differences in adhesion‐free energy between surfaces Δ*G* ∈ [−17, −21] lead to substantially different virus desorption times. Kramer's escape‐time relation,^[^ ^10^ ^]^ $\tau_{esc}\approx \left(6\pi \eta R_{v}\delta^{2}/k_{B}T\right)\;e^{-\Delta G/k_{B}T}$, for a width of the free‐energy potential well of δ ∼ 10 nm and η as the water viscosity, leads to estimates for desorption waiting times of τ_*esc*_ ∼ 7 h for MoS_2_, while just ≈6 min for SiO_2_‐plasma. Similar exponential effects lead to a substantial difference in the number of virus captured, with a factor 15 difference between surfaces. In particular, we find MoS_2_ to be the best surface for virus capture, followed by SiO_2_. In this line, we find that plasma cleaning of surfaces appears as a possible way to significantly prevent coronavirus adsorption, which may be relevant, for instance, as a pre‐treatment for hospital material. Our results indicate that O_2_ plasma‐cleaning of SiO_2_ reduces the capacity of SiO_2_ to capture TGEV, when compared to isopropanol‐cleaned SiO_2_ and the rest of surfaces under study. We also explored the use of bicomponent surfaces to sense, in a single experiment, the capacity of viral particles to attach to different surfaces. Using this approach, we find that MoS_2_ captures four more times virus than SiO_2_ cleaned with isopropanol, making this material a good candidate for applications such as air filters, mopping material or biosensors. Given the capacity of MoS_2_ to affect lipid membranes under blue light irradiation,^[^ ^67^ ^]^ it could be used as a double‐purpose material which not only captures viruses but also inactivates them. Precisely, a recent study in this direction used TiO_2_ substrates and ultraviolet light to deactivate the SARS‐CoV‐2 virus.^[^ ^29^ ^]^ Additionally, experiments with mica and mica functionalized with poly‐L‐lysine (PLL) have shown that surface functionalization can improve virus adhesion, however up to a more moderate factor of two (Table 2).

The study of the virus profiles using AFM surprisingly showed that differences in viral capture (Table 2) are not correlated to the virus height or deformation measured from the contact angle θ_0_ (1). For instance, adsorption on the MoS_2_ surface leads to less deformed virus θ_0_ ≈ 88° than on SiO_2_ cleaned with O_2_ plasma θ_0_ ≈ 55°. Yet, MoS_2_ captures ≈14 times more virus (Table 2). This behavior is certainly at odds with that found in the case of simple vesicles, whose deformation obviously increases with the adhesion energy *W*. Coarse‐grained simulations of the coronavirus and a Helfrich‐type model generalized to include spike contributions, were used to explain this surprising result. The key connection between experiments and theory was the calculation of the surface affinity via the dissociation constant *K*_*d*_ for each surface (highly correlated with the viral capture, Table 2). The relatively small differences in *K*_*d*_ ∈ [1 − 20] nM amongst surfaces could only be explained by the complementary physicochemical nature or surface‐interaction observed in the spikes and the CoV lipid envelope. For instance, MoS_2_ should present a high attraction to the CoV spikes, but a mild interaction with the virus membrane while, the opposite takes place in the case of SiO_2_‐plasma surface. As shown in Figure 7B, the counter‐correlation between the surface‐energy density of the membrane *W*_mem_ and the spike *W*_spk_ (blue and orange lines respectively) is pretty consistent when taking into account all the studied cases.

Moreover, it is interesting to highlight that Δ*G* and *K*_*d*_ for our surfaces (1 to 20 nM) are similar to the binding affinity of SARS‐CoV‐2 and SARS‐CoV spikes to ACE2 receptors whose kinetics were studied by surface plasmon resonance^[^ ^68^ ^]^ and biolayer interferometry binding analysis.^[^ ^69^ ^]^ This later work reports values of *K*_*d*_ = 1.2 nM for SARS‐CoV‐2 and 5 nM for SARS‐CoV, close to several works on umbrella sampling MD simulations,^[^ ^70^, ^71^ ^]^ precisely finding Δ*G* ≈ −20*k*_*B*_ *T* for the RBD‐ACE binding free energy. Larger values (i.e. smaller affinity) were reported for the spike‐ACE2 in other experiments^[^ ^68^ ^]^ (up to 100 nM). Taking into account that membrane‐membrane interfacial energies can be of the same order or even larger^[^ ^10^ ^]^ than surface‐membrane energies (*W* ∼ [0.1 − 10] mJm^−2^), our results suggest some nuances to the standard entry pathway picture, indicating the need of further research on the role of the virus envelope. The CoV might adsorb to the host cell membrane, signaling the initial infection step, where the membrane protein M plays an important role.^[^ ^72^ ^]^ Then, it might use several entry pathways aside from the RBD‐ACE2 receptor route. A recent contribution shows that COVID infection is possible (yet slower) even without ACE2 receptors, due to cleavage to a cell membrane protusion and activation of S2 unit via extracellular protease, leading to membrane fusion and infection.^[^ ^73^ ^]^ Membrane–membrane attraction might enhance this process. Attraction between hydrophilic surfaces can be driven by hydrogen bond formation^[^ ^32^ ^]^ and even in slightly negative charged surfaces.^[^ ^40^ ^]^ Also, divalent ions such as Ca^2+^ or minute concentrations of specific ions are well known to give rise to mutual adhesion of bilayers containing negatively charged lipids.^[^ ^10^, ^11^ ^]^ A liposome interacting with a membrane creates substantial local stress^[^ ^74^ ^]^ and might also lead to spontaneous fusion by exposure to the strong hydrocarbon hydrophobic attraction.^[^ ^10^ ^]^ Fusion, one of the critical aspects of infection, just requires the virus membrane surface tension to be larger than that of the host cell.^[^ ^12^ ^]^

Although this work is not focused on surface chemistry details, we attempt some discussion on the putative molecular origins underlying the alleged membrane and spike complementarity in terms of surface interaction. We start by assuming that the virus membrane (similar to the membrane it steals) is slightly negatively charged and of polar nature, hydrophilic and prompt to form hydrogen bonds. By contrast, at biological pH 7 (used in our experiments), the spike S1 has been reported to be positively charged in virtually all variants of CoV,^[^ ^75^ ^]^ which is quite probably related to natural evolution toward the negatively charged ACE2 receptor. The spike also presents a relatively high dipole pointing outward the S1 unit.^[^ ^76^ ^]^ This brief description already points out some degree of complementarity for the spikes and the virus membrane interactions. At the electrostatic level, mica is negatively charged, which might attract the spikes. Consistently, in mica we find one of the highest spike adhesion energies, which is similar to what has been found in quartz‐microbalance studies in mica.^[^ ^27^ ^]^ By contrast, mica‐PLL (C_5_H_12_N_2_O)‐n leads to a high positive charge, which would in principle enhance the interaction with negatively‐charged phosphate groups from lipids and repel the spikes. This interpretation is consistent with our results (Figure 6).

Our findings suggest a strong attraction of the virus membrane to SiO_2_‐plasma, which is hydrophilic (due to the so formed Si–OH groups) and probably presents a small negative charge. However, supported lipid bilayer formation upon rupture of negatively charged vesicles is readily observed in silica under small amount of positive charge^[^ ^40^ ^]^ (not in mica, unless calcium added to screen electrostatic repulsion). The possibility of hydrophilic attraction is strongly enhanced by the formation of hydrogen bonds.^[^ ^32^ ^]^ In SiO_2_‐plasma, we find a mild interaction with the spikes (Figure 7B), but the reason why is not clear, and it might be due to the negatively charged glycoproteins (apparently designed to avoid detection by negatively charged antibodies).^[^ ^77^ ^]^ SiO_2_‐isopropanol is mainly hydrophobic and neutral, so there should not be a strong attraction or repulsion to the membrane or the spike. HOPG is also very hydrophobic and neutral. Moderate attraction from both *W*_mem_ and *W*_spk_ (Figure 7) probably due to dispersion forces and hydrophobic patches in the virus envelope,^[^ ^3^ ^]^ seems to optimize virus capture. Finally, MoS_2_ is neutral and hydrophobic, which should explain the moderate membrane attraction, large contact angle and small virus deformation (Table 1). However, MoS_2_ is well known for having a strong affinity to the coronavirus spike,^[^ ^41^ ^]^ partly due to the formation of disulfide bonds with the relatively abundant cysteines in the spike.^[^ ^42^ ^]^ This explains the large spike‐MoS_2_ energy leading to the highest viral capture (Table 1). As an aside, although the RBD ‐MoS_2_ affinity is high and similar to the RBD‐ACE affinity (≈11 nM^[^ ^2^ ^]^) a recently developed nanomaterial reaches the pM range.^[^ ^2^ ^]^

The TGEV ability to adhere to disparate surfaces, added to its exceptional self‐healing properties^[^ ^16^ ^]^ surely help its adaptation to a wide range of environmental circumstances. Our study unveils that the TGEV adaptability to adhere on different surfaces is due to a seemly complementary physicochemical nature of the lipid envelope and the spikes. A relevant question is the possible extrapolation of the present findings to other members of the coronavirus family and to the SARS‐CoV‐2 in particular. Some evidence was found in comparative studies on virus adsorption on the solid phase of wastewaters where, at ambient temperature, the coronavirus (SARS‐CoV‐2) was found to present the highest adhesive strenght amongst all virus considered (either enveloped or non‐enveloped).^[^ ^21^ ^]^ The multidisciplinar protocol presented hereby is generally applicable to other viruses and in particular could be used to investigate adsorption of human viruses such as the SARS‐CoV‐2. However, it is possible to extrapolate some of the conclusions of this work. Envelopes of all coronaviruses are taken from the host cell, so they should not present strong differences amongst species. Details of the spike charge distribution vary amongst coronavirus and might lead to different adhesion behavior, particularly on those surfaces where the spike‐surface attraction is dominant and of electrostatic type (such as on negatively charged mica). However, minor differences would be expected in other surfaces (such as MoS_2_) where the spike adsorption is dominant due to the formation of disulfide bonds with cysteines, abundant in CoV spikes.^[^ ^42^ ^]^

This adaptability and versatility of the coronavirus family might be the reason why three coronaviruses have already crossed the species barrier to cause deadly pneumonia in humans since the beginning of the 21st century: SARS, Middle‐East respiratory syndrome coronavirus (MERS‐CoV), and SARS‐CoV‐2. The multidisciplinary single‐particle methodology proposed here might help in finding potential therapies or prophylactic alternatives.

## 3 Experimental Section

### Virus Purification, Cell Cultures, Buffers

TGEV virions were purified using a slightly modified version of a previously reported method^[^ ^14^ ^]^ Initially, supernatants from TGEV‐infected cells were clarified by centrifugation at 4500 × g for 20 min at 4 °C. Virions were then sedimented by ultracentrifugation at 112 000 × g for 120 min through a 31% (w/w) sucrose cushion in TEN buffer (10 mM Tris‐HCl [pH 7.4], 1 mM EDTA, 1 M NaCl) with 0.2% Tween 20 (Sigma, Saint Louis, MI, USA). The viruses were subsequently centrifuged through a continuous 15% to 42% (w/w) sucrose gradient in order to separate wild‐type virions and defective virions with lower density. Finally, virions were pelleted by ultracentrifugation at 112 000 × g for 60 min and resuspended in TNE buffer (10 mM Tris‐HCl [pH 7.4], 1 mM EDTA, 100 mM NaCl) using 0.05% Tween 20. Purified virions were disaggregated by sonication (six pulses at medium intensity in a Branson sonifier 450). SDS‐PAGE protein analysis of the purified TGEV virions using Coomassie staining showed the presence of the major viral proteins S, N and M. This result confirmed the presence of TGEV virions in the samples used for AFM and cryogenic electron microscopy.

### Virus Genome Mass and Bulk Concentration

Taking into account that the average molecular weight of ribonucleotide triphosphates is 500 g mol^−1^ or 500 Da and that the average weight of a Dalton is 1.66054 10^−24^ g, we calculate the weight of the TGEV strain PUR46‐MAD consisting of 28 580 nucleotides^[^ ^78^ ^]^ obtaining a final result of 2.37 × 10^−17^ g per copy, used to calculate the virus concentration from the total genome mass. The sample was analyzed after a 1‐h treatment at 37 °C with 1% SDS and 0.5 mgmL^−1^ of proteinase K in TNE buffer and subsequently measured on a NanoDrop 1000 (ThermoFisher) to obtain the concentration of RNA in our sample. Thus, the achieved viral titer of the stock was (3.56 ± 0.03) × 10^12^ virus/mL.

### AFM Imaging and Profile Correction

Measurements were performed with an AFM (Nanotec Electrónica S.L., Madrid, Spain) in Jumping Plus Mode^[^ ^79^ ^]^ In this mode, lateral interactions were minimized as the lateral displacement was done with the tip far from the sample. The tip moved in the Z axis (perpendicular to the surface) until a certain present interaction force. Topography data was obtained and the tip released the surface and is moved laterally one pixel to repeat the procedure again. Parameters for the Jumping Plus Mode were: jump off = 100 nm, jump sample = 200 nm and control cycles = 20. Image size was typically 8 × 8 microns, but some images of smaller size were taken for each sample, in order to check viral integrity. All the experiments were carried out at pH 7.

AFM tips used were NANOWORLD^*TM*^ PNP‐DB‐20, with a tip radius below 10 nm. Cantilevers used presented a nominal force constant of 0.06 N/m (200 × 40 nm rectangular cantilevers). The applied perpendicular force for Jumping Plus imaging was calculated precisely with Sader's method^[^ ^80^ ^]^ for rectangular cantilevers, and it was in the 50–100 pN range, in order to nullify the impact of the loading force on viral height, as the deformability of lipid‐enveloped viruses was reportedly higher than protein‐capsid ones^[^ ^14^, ^81^ ^]^

The topography profiles needed to be corrected for the dilation induced by the AFM tip radius *R*_AFM_ and the compression due to the applied force. The protocol is illustrated in Figure 2A. An obstacle located at some given position *x* is sensed by the AFM at *x*_*AFM*_ = *x* ± *R*_*AFM*_. Therefore, for an object of width 2*L* the expansion factor in any plane direction (x) is λ_*x*_ = 1 + *R*_*AFM*_/*L*. Experimental values were ≈λ_*x*_~ ≈ 1.4, which agree with Ref. [82]. The compression in the vertical direction was related to the particle stiffness (*k*_*sp*_ ≈ 0.01 Nm^−1^).^[^ ^14^ ^]^ For 100 pN force, this implies a deformation δ_*y*_ = *F*/*k*_*s*_ ∈ [5 − 10] nm. Thus, the compression factor was λ_*y*_ ≈ (*H* − δ_*y*_)/*H*, where *H* is the virus height directly measured from the AFM profile (Figure 2A), resulting in λ_*y*_ ≈ 1.1. Using this information, the shape of the AFM profiles were corrected by the re‐scaling *y*(*x*) = λ_*y*_ *y*_*AFM*_(*x*/λ_*x*_).

### Viral Capture on Surfaces and Dissociation Constant Calculation

Viral Capture (VC) is defined as:

(3) $$\text{VC}=\frac{\text{Number of viruses scanned}}{\text{Total scanned area}}$$

where the total scanned area is given in µm^2^. Thus, VC represents the number of virus detected by AFM in 1 µm^2^. VC is defined for a given virus concentration and surface, by analizing a sample of *n* = 6 different images from which VC is obtained and its error bar (arithmetic mean and standard error). For bicomponent samples with two different regions, VC is defined for each region by dividing the number of viruses in the region by the area (in µm^2^) of the region. Note that VC can be further divided by the concentration of viruses (in nM) for each case, therefore grouping all dilutions and concentrations in a single combined value that defines the capture capacity for each substrate globally.

Binding affinity was quantitatively evaluated via the dissociation constant *K*_*d*_ following the Hill–Langmuir equation for non‐cooperative binding^[^ ^61^ ^]^

(4) $$\Theta =\frac{\Theta_{max}{\left[V\right]}_{F}}{K_{d}+{\left[V\right]}_{F}}$$

where Θ stands for the area fraction covered by viruses which is set to Θ = VC π*L* ^2^, with *L* the virus surface‐projected radius (see Figure 3); [*V*]_*F*_ is the concentration of free viruses. *K*_*d*_ represents the virus concentration required for a half‐coverage of the surface. It is assumed that the situation is in the Henry regime (far from saturating conditions) *K*_*d*_ ≫ [*V*]_*F*_, which is later confirmed by the results. The maximum virus coverage Θ_*max*_ is set to 0.64 which roughly equals the 2*D* random packing limit. Equation (4) simplifies to

(5) $$\Theta =\Theta_{max}\frac{{\left[V\right]}_{F}}{K_{d}}$$

Note that [*V*]_*F*_ = [*V*]_0_ − [*V*]_*C*_ where [*V*]_0_ is the initial virus concentration in the sample and [*V*]_*C*_ the correction due to the virus captured onto the substrate, estimated as

(6) $${\left[V\right]}_{C}=\frac{\mathrm{VC}\;\pi R_{s}^{2}}{N_{A}V}$$

where VC is the virus surface density, R_*s*_ ≈ 4 × 10^3^µ*m* the 30 µL droplet projected radius (average over surfaces) and V is the sample's droplet volume 3 × 10^−5^ L (equal for every case). *N*_*A*_ = 6.022 × 10^23^ mol^−1^ the Avogadro number. Note that [*V*]_*C*_ ≪ [*V*]_0_ and this correction can be neglected.

As explained in the main text (see also Equation (5)) *K*_*d*_ = Θ_max_/(*a*π*L* ^2^), where *a* the slope of the lines in Figure 4. The relative error for *K*_*d*_ is then Δ*K*_*d*_/*K*_*d*_ = Δ*a*/*a* + 2Δ*L*/*L* which results to be Δ*K*_*d*_/*K*_*d*_ ≈ 0.15. As *K*_*d*_ = exp [Δ*G*/*k*_*B*_ *T*], this also determines the error in free energy Δ[Δ*G*/*k*_*B*_ *T*] = Δ*K*_*d*_/*K*_*d*_ ≈ 0.15.

### Image Processing, Virus Counting and Viral Height Measurements

Images were processed using WSxM program (www.wsxm.es).^[^ ^83^ ^]^ First, the function “flatten plus” was used to help with the flattening process for images and drift correction. Second, images the function “equalize” was used to adequately fix the “zero” level of the sample. Then, the function “flooding” was applied for image quantification. To quantify the number of viruses, flooding was set to “find hills” with a “35 nm” limit. The number of detected “hills” equals the number of virus detected, the value was then used for VC calculation in Equation (3). Then, for viral height analysis, the same “hills” information was copied to a spreadsheet, were the maximum heights were plotted, using SigmaPlot 11.0 (Systat Software Inc.), as height histograms, later normalized and linearized using the same software for a direct comparison among samples.

### Sample Preparation and Virus Addition

Muscovite mica and highly oriented pyrolytic graphite (HOPG) were prepared by stack‐exfoliation (freshly cleaving). Poly‐L‐lysine functionalization of mica was achieved as previously described^[^ ^14^ ^]^ Briefly, a 30 µL droplet of 0.1% poly‐L‐lysine was deposited onto freshly cleaved mica for 15 min and rinsed with 1 mL ultra‐pure water. Then, a highly pure stream of N_2_ was applied to the surface to dry it. This method results in a thin layer of poly‐L‐lysine deposited onto the mica.

For SiO_2_ surfaces, the method can be described as follows. Starting from a 4' Si/SiO_2_ wafer (300 nm) from MicroChemicals GmbH (Ulm, Germany), square substrates of 10 mm lateral size were cut with a diamond tip. They were then cleaned in an ultrasonic bath of isopropanol for 15 min and subsequently blown with nitrogen. This resulted in the surfaces dubbed as SiO_2_‐isopropanol. For the preparation of some of the samples (dubbed as SiO_2_‐plasma) an oxygen plasma surface treatment at 50% power for 15 min using a Femto Low Pressure plasma system from Diener Electronic GmbH (Ebhausen, Germany) was performed.

Regarding virus addition, the same procedure was applied for every substrate: a 30 µL droplet with a viral titer of (3.56 ± 0.03) × 10^12^ virusmL^−1^ in TNE buffer (or accordingly diluted by the desired dilution factor) was incubated for 60 min and rinsed with a clean buffer 5 times, adding 1x incubation volume and removing it. A final volume of 90 µL was then used for measuring the virus under the AFM.

### MoS_2_ Flakes Preparation

A single‐zone tube furnace was used for the growth of MoS_2_. 2.5 mg MoO_3_ powder was evenly distributed in an alumina boat and located at the center of the furnace. A layer of molecular sieve (Alfa Aesar 5A 1–2 mm diameter pellets) covered the MoO_3_ boat to control the growth rate. SiO_2_/Si substrates, spin‐coated with 0.5 mg mL^−1^ NaOH promoter, were placed on the MoO_3_ boat. 60 mg sulphur powder in another alumina boat was located 17 cm upper stream at the edge of the furnace. Before starting growth, the tube was purged with 460 sccm N_2_ for 20 min. The N_2_ flow rate was then decreased to 60 sccm. The temperature of MoO_3_ was set and kept at 720 °C for ≈10 min, while the temperature of sulphur could reach 230 °C. At the finishing step, the furnace was switched off, the sulphur source was pulled out of the heating zone, and the MoO_3_ boat was let to cool down in 460 sccm N_2_ gas.

### Coarse Grained Model

To conduct in‐depth computational simulations of virus‐surface interactions, a coarse‐grained model is devised that combines a modified virus spike protein model^[^ ^52^ ^]^ with a single‐bead coarse‐grained membrane model.^[^ ^84^ ^]^ Full details of the model, including parameters and several validation tests, are available in the Supporting Information. The virus lipid envelope was modeled as a single layer of “polar” beads, which allows for the fine‐tuning of critical membrane properties such as 2D diffusion, mean radius, and bending rigidity. The chosen parameters create a fluid membrane composed of beads ≈1.8 nm in radius, resulting in a bending rigidity of κ = (39 ± 1)*k*_*B*_ *T*, as ascertained from thermal fluctuations.

The spike proteins were represented by a network of 17 elastic blobs, with the initial blob also forming part of the membrane. This integration guaranteed a smooth membrane‐spike coupling and sets the spike's diffusion mobility in line with values reported in Ref. [56]. The model further includes angular bonds within the spike structure to maintain the orientation of its various sections.

A critical element of the model was the differentiated surface interaction potentials for the membrane and spike proteins. While each bead, whether part of the membrane or a spike, interacted with the surface through a Lennard–Jones‐like potential, the parameters of this potential could be independently adjusted for the membrane and spike beads. This versatility allowed for the simulation of a range of adhesion behaviors, from purely repulsive to attractively adhering scenarios, depending on the nature of the virus‐surface interaction.

The model, with ≈2000 beads, strikes a balance between detail and computational efficiency. An NVT overdamped Langevin integrator was employed to effectively manage polar interactions and particle orientation updates. This methodological choice is pivotal for the extensive simulations required to explore the virus adhesion process in depth.

### Umbrella Sampling Surface

Umbrella sampling^[^ ^85^ ^]^ was used to obtain the free energy profile for the interaction between the CG virus model and the surface. Umbrella potentials at each window centered at *z*_*i*_, consist of harmonic potentials applied to the vertical coordinate of the center of mass of the virus envelope *z*_*cm*_ (i.e. excluding the spikes), i.e., (*k*/2)(*z*_*i*_ − *z*_*cm*_)^2^. Values of *z*_*i*_ separated by ≈5 nm were swept over to generate a series of overlapping biased probability “windows” or “umbrellas” from which the less‐biased free energy profile $\Delta F(z_{cm})$ was obtained, using the weighted histogram analysis method (WHAM).^[^ ^62^ ^]^

### Virtual AFM Image

In the process of generating virtual Atomic Force Microscopy (AFM) images from the coarse‐grained simulations, the first step involves positioning a virus on a surface using the surface interaction set by ε_*spk*_ and ε_*mem*_ and run a long Brownian dynamics simulation to let the system reach the equilibrium (total energy, height). To accurately replicate Atomic Force Microscopy (AFM) the bead positions during the data acquisition phase was fixed. To imitate real‐life conditions, the spike proteins that do not make direct contact with the surface were discarded, a scenario that mimics their displacement by the AFM tip in an actual AFM scan. The resulting AFM images represents the locus of the beads associated with the lipids and those spike proteins that are in immediate contact with the surface. The radius of the AFM tip was then defined, typically (set to ≈15–30 nm). The resolution of the image was then determined, frequently being either 64 × 64 or 128 × 128 pixels. This resolution allowed to individually navigate through each pixel in the x‐y plane. For each pixel, the height as the point of collision between the AFM tip and some virus blob was ascertain, or the surface. By mimicking the functionality of real‐world AFM, a detailed topographical image of the sample was generated. This virtual AFM image can be utilized for subsequent analysis (see Figures 1c and 3), providing with valuable microscopic insight, upon comparison with experimental data.

### Statistics

The analysis of Langmuir isotherms was carried out by counting adsorbed virus in *n* = 6 different images (*n* = 10 for Mica) at each virus concentration and surface. For the five concentrations analyzed, this yields a sample of *n* = 30 images from which the dissociation constant *K*_*d*_ is obtained. In the case of indentation profiles the samples of *n* = 20 profiles for each surface were typically used. The error bars for the scaled radius [*H*/*R*_*v*_] correspond to the standard error of the ratio for each individual virus, over these *n* = 20 samples. The overall average radius of the virus *R*_*v*_ and its error bar was obtained from the sample of indentations over all surfaces *n* ≈ 100.

## Conflict of Interest

The authors declare no conflict of interest.

## Author Contributions

A.B.G‐A. and P.I.‐F. equally contributed to this work. R.D.‐B. and P.J.P. handled the conceptualization; P.I.‐F., A.G.‐A., and R.D.‐B. managed data curation; R.D.‐B. conducted the formal analysis; P.J.P., J.M.‐B., and R.D.‐B. secured funding; A.G.‐A. investigated TGEV, P.I.‐F. worked on the CG model, and M.C. focused on HCIV‐1; methodologies were developed by R.D.‐B., A.G.‐A., P.I.‐F., and J.M.‐B.; project administration was overseen by P.J.P. and R.D.‐B.; resources were provided by G.L.‐P. (SiO2), H.Y., Y.W., S.S., M.Ch., and P.A. (MoS2), H.M.O. (HCIV‐1), and J.M.‐B. (TGEV); software was handled by P.I.‐F. and P.P.‐A.; supervision was provided by R.D.‐B.; validation was conducted by R.D.‐B., A.G.‐A., and P.I.‐F.; visualization was carried out by R.D.‐B., P.I.‐F., and P.J.P.; the original draft was written by R.D.‐B., and the review and editing were performed by R.D.‐B., P.J.P., A.G.‐A., and P.I.‐F.

## Supporting information

Supporting Information

Supplemental Movie 1

Supplemental Movie 2

Supplemental Movie 3

## Data Availability

The data that support the findings of this study are available in the supplementary material of this article.
