# Supplementary material for: Broad Adaptability of Coronavirus Adhesion Revealed from the Complementary Surface Affinity of Membrane and Spikes
Source: Adv Sci (Weinh). 2024 Sep 4;11(41):2404186. doi: 10.1002/advs.202404186 (PMC11538687; doi:10.1002/advs.202404186)
Supplement: Supplementary file 1 — Supporting Information [file ADVS-11-2404186-s001.pdf]

ADVANCED SCIENCE
Open Access

Supporting Information

for *Adv. Sci.*, DOI 10.1002/advs.202404186

Broad Adaptability of Coronavirus Adhesion Revealed from the Complementary Surface Affinity of Membrane and Spikes

*Aritz B. García-Arribas, Pablo Ibáñez-Freire, Diego Carlero, Pablo Palacios-Alonso, Miguel Cantero-Reviejo, Pablo Ares, Guillermo López-Polín, Han Yan, Yan Wang, Soumya Sarkar, Manish Chhowalla, Hanna M. Oksanen, Jaime Martín-Benito, Pedro J. de Pablo and Rafael Delgado-Buscalioni**

Supplementary Materials
Broad adaptability of coronavirus adhesion revealed from the complementary surface affinity of membrane and spikes

Aritz B. García-Arribas,[1†] Pablo Ibáñez-Freire,[2†]
Diego Carlero,[3] P. Palacios-Alonso,[2]
Miguel Cantero-Reviejo,[1]
Pablo Ares,[1] Guillermo López-Polín[1],
Han Yan,[4] Yan Wang,[4]
Soumya Sarkar,[4] Manish Chhowalla,[4]
Hanna M. Oksanen,[5] Jaime Martín-Benito,[3]
Pedro J. de Pablo,[1,6] Rafael Delgado-Buscalioni[2,6*]

Virus morphology

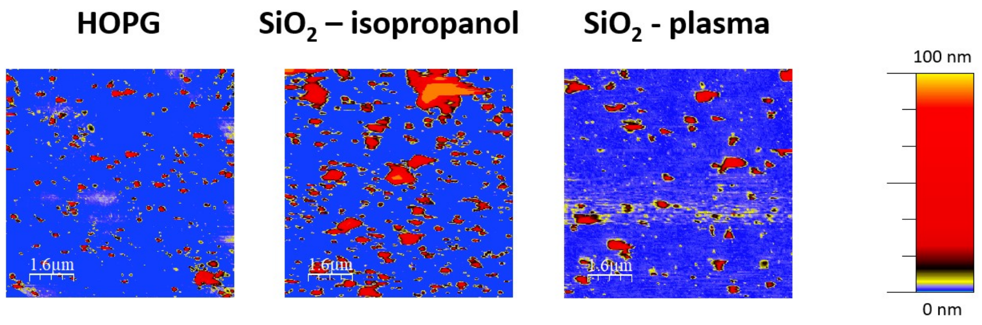

Figure S1: AFM imaging of TGEV on mica, mica + PLL and HOPG at different dilutions.

AFM size estimation based on the virus width indicates adsorbed spikes

There are several ways to estimate the particle radius from AFM profiles. As stated in the main text, we used a volume-based method, namely

$$\frac{4\pi}{3}R_v^3 = \mathcal{V} = \frac{1}{2}\int_{x_0}^{x_1} \pi y(x)^2 dx \qquad (1)$$

where $\mathcal{V}$ is the volume of the spherical virus. But a more commonly used method consists on assuming that i) the area is not strongly modified by adhesion and ii) approximate the particle shape to that of a drop of radius R_s. This yields $4\pi R_s^2 \approx 2\pi R_c H + \pi L^2$, where $R_c = H/2 + L^2/(2H)$ is the effective curvature radius of the viral envelope,(see Fig. 3B). The radius estimated from this *constant surface* assumption is then,

$$R_s = \left(\frac{H^2 + 2L^2}{4}\right)^{1/2} \qquad (2)$$

The analysis of the virtual AFM profiles of the CG CoV model reported similar radius obtained from the volume-based Eq.1 and the surface-based radius, $R_v \approx R_s \approx 45$nm, consistent with the real radius of the CG model 41 nm (taking into account that the

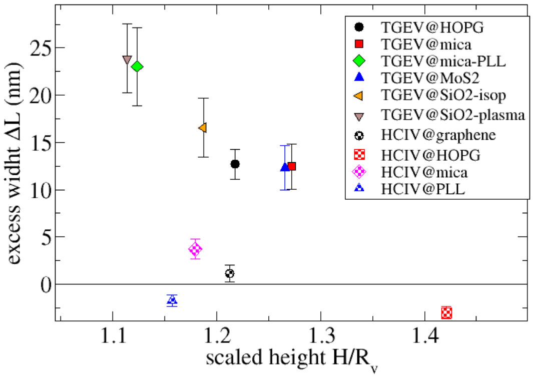

Figure S2: Excess width ΔL with respect that of adsorbed liposome with fixed area (see text) against the scaled height H/R_v for TGEV and HCIV-1 vesicles in different types of surfaces. The average of ΔL over surfaces is virtually zero 0.75 nm for HCIV-1 shells, while for the TGEV coronavirus is about 15 nm. Error bars correspond to the standard error $(\text{Variance}/N_{samples})^{1/2}$.

building CG-beads have 4 nm in diameter). In the case of experimental HCIV-1 vesicle profiles we obtain $R_v = (29 \pm 2)$ nm and $R_s = (31 \pm 2)$ nm; both values being consistent to that measured by chromatography (33 ± 2) nm. But unexpectedly, in the coronavirus case, the constant-area relation (Eq. 2) lead to a larger radius $R_s = (55 \pm 2)$ nm. A more detailed analysis revealed that this systematic difference between R_s and R_v contains information. The surface-based estimation R_s is very sensitive to the particle width L, obtained from AFM, and L is strongly affected by the presence of tethered structures such as the TGEV spikes. 2D AFM images (Fig. 1A and B) usually presents protrusions which *might* indicate adsorbed spikes. In the case of Fig. 1C we are certain of this fact, as it corresponds to the coarse-grained model. To validate this hypothesis we investigate the statistics of the difference between R_v and R_s in the TGEV particle.

To that end we compared the AFM width L (corrected from tip expansion) with the "average" width $\bar{L}_s = (2\bar{R}^2 - H^2/2)^{1/2}$ associated to the average of both radii $\bar{R} =$

$(R_s + R_v)/2$ and to Eq. 2. For a drop-like shape one expects $L \approx \bar{L}_s$ and this is in fact the trend observed for the HCIV-1 shell ($\Delta L = L - \bar{L}_s = (2 \pm 2)$ nm). For the TGEV particle, we get $\langle \Delta L \rangle = (17 \pm 2)$nm when averaged over all substrates. Notably, a more detailed analysis of the data reveals that this "excess width" ΔL decreases with the TGEV virus height H (Fig. S2). This result suggests that this excess in L is related to the presence of close-by spikes, probably adsorbed to the surface. In other words, CoV particles present a larger spike exposure over the plane (larger ΔL) as they get more adsorbed and deformed near the surface (smaller H).

Averaged AFM profiles

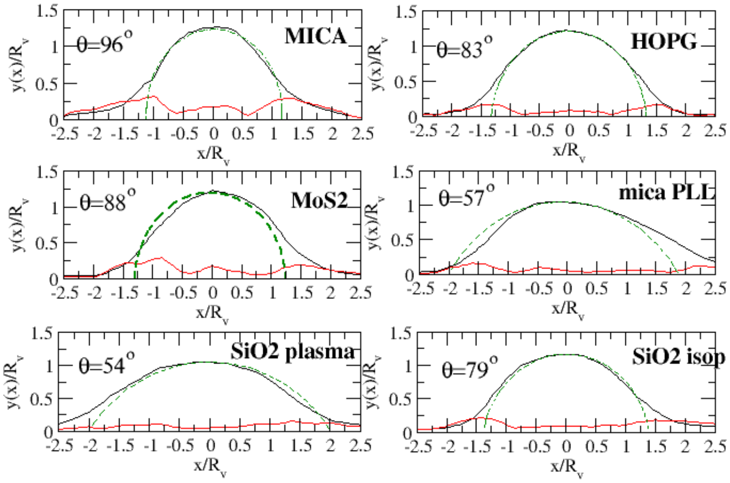

Figure S3: Average AFM profiles obtained from a set of ca. 20 AFM images per surface. The axis are normalized with the virus radius evaluated for each particular profile using the volume method in Eq. 1. Dashed lines are the spherical-cap approximation to the profile, also indicating the contact angle θ. The red lines indicate the standard deviation at each coordinate Std$[y](x)$.

Surface	H/R	Contact angle	WR^2/κ
HOPG	1.42 ± 0.07	105 ± 10	4.0 ± 0.5
Mica-PLL	1.0 ± 0.1	65 ± 5	6.0 ± 0.5
Mica	1.3 ± 0.1	75 ± 5	5.2 ± 0.2
Graphene	1.22 ± 0.07	65 ± 5	6.2 ± 0.2

Table S1: Experimental results for the HCIV-1 vesicle for the scaled height H/R, contact angle and derived non-dimensional adhesion energy density. Results correspond to averages from a set of 25 AFM images on each surface.

Results for the HCIV-1 vesicles on different surfaces,obtained from about 25 AFM averaged profiles on each surface, are shown in Table S1.

Bicomponent surfaces: MoS_2 and SiO_2-isopropanol

Bicomponent substrates (i.e., surfaces made of two different materials, each one confined to specific areas) were put to the test to improve our understanding of viral adhesion in the presence of two well-differentiated materials. For this, we used MoS_2 flakes on SiO_2 surfaces (Fig. S4).

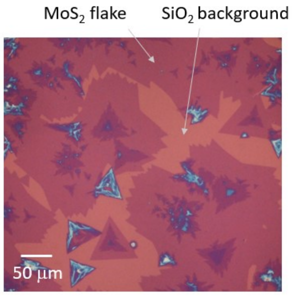

Figure S4: Optical microscopy image of MoS2 flakes on SiO2 background. The image confirms that monolayer MoS2 flake boundaries are defined by irregular sharp angles.

AFM imaging revealed a clear preference of TGEV towards MoS_2 flakes (Figure S5), identifiable as linear boundaries in the background with an increase in height. A control without virus can be checked in Figure S5. Holes (black circles) can be distinguished in

both areas (flake and non-flake), which are caused by the sample preparation treatment. These do not affect the viral quantitation process (neither in VC, nor in height measurements), as images are later equalized (Fig. S5, right panel) to establish the out-of-flake background as a zero-height reference.

Experiments were performed in a 7-day lapse of time to check if any changes occurred in viral quantitation, which was not the case. Viral dilutions under use were 1/30 and 1/100, with the latter resulting in a much better reproducible as viruses are more loosely distributed, enabling a more efficient counting of particles. As a result, 1/100 samples exhibit less relative error in VC values (Fig. S6). VC values for each dilution were: for 1/30: on flake VC = 12.54 ± 6.59 and out of flake VC = 3.52 ± 2.18; while for 1/100: on flake VC = 2.90 ± 0.92 and out of flake VC = 0.73 ± 0.25.

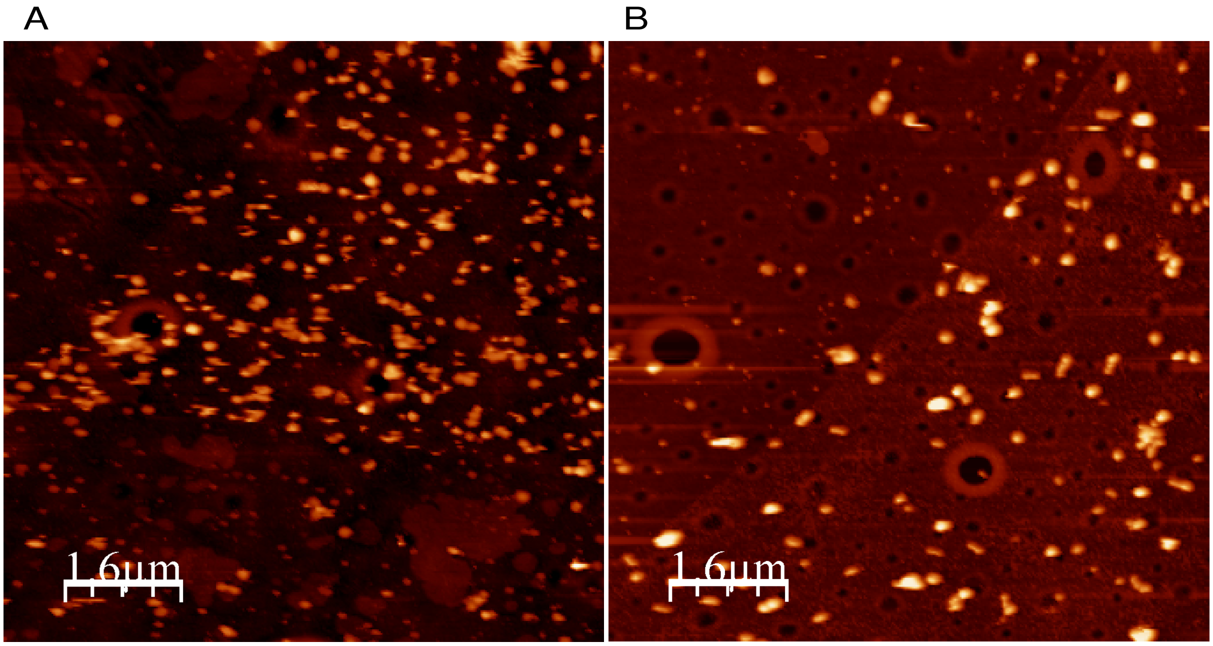

Figure S5: Representative AFM images of boundaries between MoS_2 flakes and the SiO_2 background. Viral particles show a clear preference towards the MoS_2 flake. Holes appear as a result of flake preparation process through CVD. Viral dilution used: 1/30 for A, 1/100 for B. Image size = $8 \times 8\ \mu m^2$.

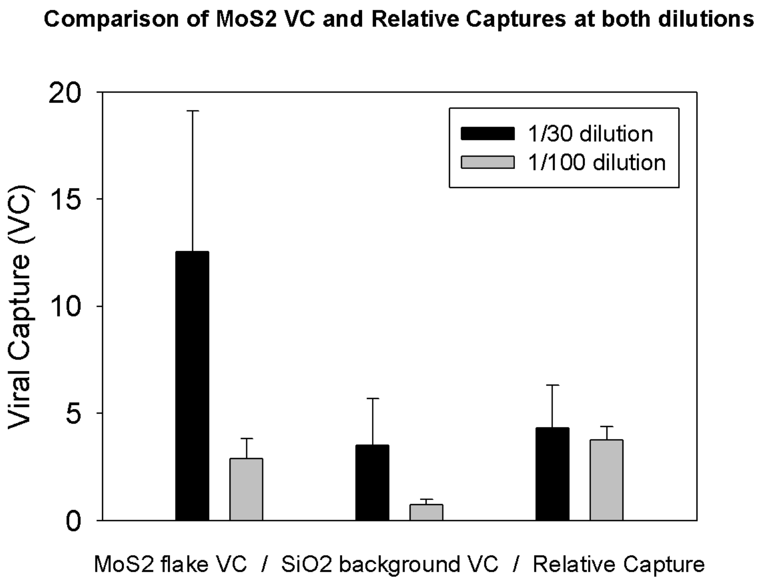

Figure S6: Absolute VC and relative capture (VC_{Flake} / $VC_{Background}$) of MoS_2 flakes and SiO_2 background at both viral dilutions. A t-test performed between the two relative capture values gives p = 0.358, therefore a non-significant difference.

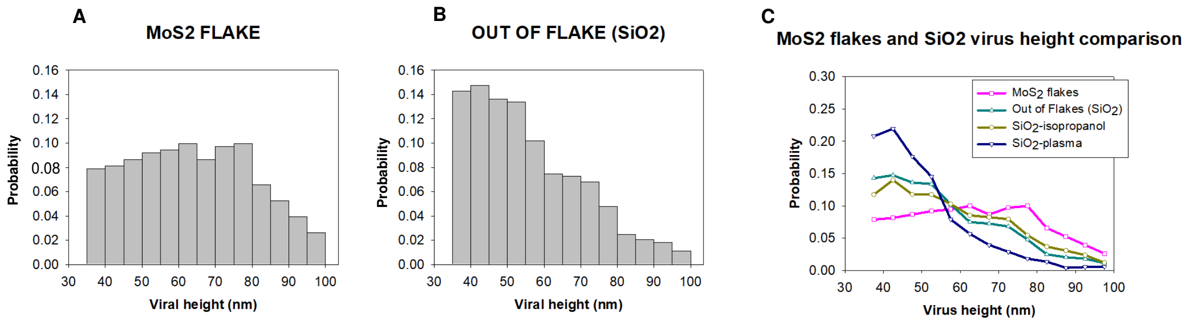

Figure S7: Normalized histogram heights of (A) virus on MoS_2 flakes and (B) out of flakes SiO_2 background. (C) shows the merged height histogram profiles of both samples in comparison with SiO_2 samples (isopropanol- and plasma-cleaned. MoS_2 flakes show a comparatively higher amount of larger viruses, while the height profiles of out of flake SiO_2 background and SiO_2-isopropanol show similarity.

Figure S6 shows a comparison of both data sets, and results seem logical: adding 3.3 times more viruses results in a ~4-fold increase in absolute VC, which is reasonable considering the great deviation for absolute VC values for the 1/30 sample. Moreover,

the combination of both data sets is acceptable, as a t-test of relative capture values gives $p = 0.358$. Considering all data as a single sample (regardless of the dilution), it reveals a relative capture, defined as VC_{Flake} / $VC_{Background}$, of 4.17 ± 1.32 (n = 20 total images of different areas with the simultaneous presence of both materials), which means that approximately 4 viruses attach to MoS_2 flakes for each virus deposited onto the SiO_2 background, regardless of the viral dilution used. Furthermore, VC values for out-of-flake SiO_2 are comparable to those from SiO_2-isopropanol.

Viral height was analyzed, and normalized histograms are shown in Fig. S7A and S7B, with a direct comparison in Fig. S7C. Results clearly indicate that the relative amount of large particles is significantly higher on MoS_2 flakes than out of them. In addition, the out-of-flake SiO_2 background also resembles isopropanol-cleaned SiO_2 in terms of viral height (Fig. S7C). Mean viral heights were 63.83 nm for MoS_2 flake and 55.80 nm for out-of-flake regions, the latter being close to the value for isopropanol-cleaned SiO_2 (56.77 nm, as already mentioned).

Coarse grained model

The coarse-grained virus model we use in this study is adapted to meet the specific questions raised hereby, which required taking into account the spike and membrane adhesion, mobility and flexibility. To this end, we merged two existing models: the membrane envelope is based on a single-bead membrane layer (formed with beads with polar interaction) [1] and the spikes are modeled by an elastic-network with specific angular bonds to fit with experimentally estimated spike fluctuations [2]. The objective was to maintain key physical properties while reducing unnecessary computational complexity.

Virus spikes

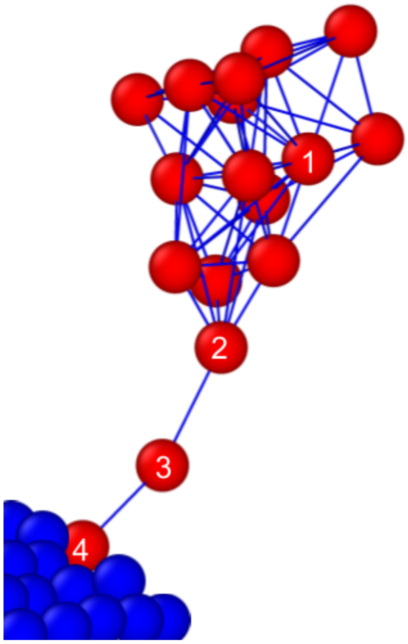

Figure S8: Schematic representation of the coarse-grained spike protein model. The model consists of 17 beads, symbolized by red spheres, which maintain their positions to preserve the overall shape and size of the spike protein. The beads are connected by blue lines representing an elastic network. The numerical labels 1 to 4 illustrate specific beads that play a crucial role in the protein's 'hip' and 'knee' regions, where angular harmonic bonds are applied to simulate realistic flexibility informed by cryo-electron microscopy data. Bead 4, in particular, serves a dual function: it is part of the protein and the membrane, it represents the segment of the spike embedded within the lipid membrane.

Our work simplifies a previous developed spike protein model [2]. This original model, based on the shape-based coarse-graining (SBCG) [3], provides a comprehensive representation of the spike protein structure. We have streamlined the detailed molecular interactions of the model, replacing them with a more generalized elastic network. This refinement aims to balance the fidelity of the original detailed structure with the practicalities of computational efficiency, ensuring that our adapted model remains both accurate and more manageable for simulation tasks.

This alteration is enough to maintaining the general shape and size of the spike protein, a key factor in its adhesion capabilities, while easing the computational demand. We keep the number of beads, 17, and its position unchanged.

In constructing the elastic network, we established a cutoff distance of $R_{\text{cut}} = 7\text{nm}$ and uniformly applied a spring constant of $K = 10\,\text{kcal/mol}\cdot\text{nm}^{-2}$, a typical value at this level of coarse graining [2]. Therefore, for every pair of beads within 8 nm of each other in the reference configuration [1], an interaction is introduced as follows:

$$U = \frac{1}{2}K(R - R_0)^2 \tag{3}$$

where R_0 denotes the inter-bead distance in the reference structure.

Furthermore, in order to accurately represent the dynamic properties of the spike protein, we integrated two angular bonds within the model. These bonds are substantiated by data derived from cryo-electron microscopy studies [4], thus imparting a realistic degree of flexibility to the protein's structure. The model employs a harmonic angular potential, represented by the equation:

$$U = \frac{1}{2}K(\Theta - \Theta_0)^2 \tag{4}$$

In this context, the parameters K and Θ_0 were determined through fitting to the experimental data. Our analysis yielded a value of $K = 11.92\,\text{kcal/mol}\cdot\text{rad}^{-2}$ and $\Theta_0 = 23.76\,\text{rad}$ for the 'hip' (comprising the first three particles of the spike, beads 4,3,2 in S8). Additionally, for the 'knee' (encompassing the second and third beads of the spike, along with another bead at the spike's head center,beads 3,2,1 in S8), the values identified were $K = 8.38\,\text{kcal/mol}\cdot\text{rad}^{-2}$ and $\Theta_0 = 1.62\,\text{rad}$.

As a result, our model is constructed with 17 coarse-grained beads interconnected by both harmonic and angular harmonic bonds. This configuration provides a practical and

effective framework for exploring the behavior of virus spikes during adhesion, striking a balance between capturing essential structural characteristics and ensuring computational tractability.

Virus membrane

The membrane is formed by a single layer of beads or blobs equipped with a polarity vector, which self-assembly in a roughly hexagonal, yet mobile, lattice. The interactions between two neighbour beads of the membrane is described by a potential energy $U(\mathbf{r_{ij}}, \mathbf{n_i}, \mathbf{n_j})$, which depends on the relative position between both beads, $\mathbf{r_{ij}}$, and the vectors $\mathbf{n_i}$ and $\mathbf{n_j}$ that describes the orientation of the beads,

$$U(\mathbf{r_{ij}}, \mathbf{n_i}, \mathbf{n_j}) = \begin{cases} u_R(r) + [1 + \phi(s)]\varepsilon & \text{if } r < r_b \\ u_A(r)\phi(s) & \text{if } r_b < r < r_c \\ 0 & \text{if } r_c < r \end{cases} \tag{5}$$

Where $r_b = \sqrt[6]{2}\sigma$, $r_c = 2.6\sigma$ with, σ being the diameter of each bead and ε is the energy unit in the model and s is an abbreviation of $(\mathbf{\hat{r}_{ij}}, \mathbf{n_i}, \mathbf{n_j})$. The function $u_R(r)$ is a 4-2 Lennard-Jones function,

$$u_R(r) = \varepsilon \left[\left(\frac{r_b}{r}\right)^4 - 2\left(\frac{r_b}{r}\right)^2 \right], \; r < r_b \tag{6}$$

$u_A(r)$ is an attractive function of the form,

$$u_A(r) = -\varepsilon \; cos^{2\zeta} \left[\frac{\pi}{2} \frac{r - r_b}{r - r_c} \right], \; r_b \langle r < r_c \tag{7}$$

here ζ is a parameters that determines the slope of the attractive region of the potential. Finally the function $\phi(s)$, contains the orientational effects of the potential,

$$\phi(s) = 1 + \mu[a(s) - 1] \tag{8}$$

being μ another free parameter of the model that tunes the diffusion and the rigidity of the membrane and,

$$a(s) = (\mathbf{n}_i \times \hat{\mathbf{r}}_{\mathbf{ij}}) \cdot (\mathbf{n}_j \times \hat{\mathbf{r}}_{\mathbf{ij}}) - sin(\theta_0)(\mathbf{n}_j - \mathbf{n}_i) \cdot \hat{\mathbf{r}}_i j - sin^2(\theta_0) \tag{9}$$

Here θ_0 is the angle between the vectors $\mathbf{n}_i$ and $\mathbf{n_j}$ that minimizes the energy, it tunes the curvature of the membrane.

The adjustment of the model parameters has been performed by searching for a combination that results in a membrane with similar size, 2D diffusion coefficient, and bending rigidity to typical values reported for SARS-CoV-2. The parameters found to match these typical values are: $\varepsilon = 5k_BT$, $\mu = 3$, $\zeta = 7$, $\theta_0 = 0$, $\sigma = 1.8\,\text{nm}$, and N = 1501 particles. The equilibrium angle used is 0, which would result in a planar surface. However, if the initial bead positions form a sphere, the simulation will result in a quasi-spherical membrane with some surface tension.

The surface corresponding to each bead is approximately $A_{bead} \approx 1250\ \text{Å}^2$, and the typical surface of a phospholipid is $A_{lipid} \approx 35.5\ \text{Å}^2$, so each bead in the model corresponds to approximately 35 phospholipids.

The mean diameter of the membrane in the simulations has been determined by projecting the surface of the membrane at different moments of the simulation over the spherical harmonic $Y_l^m = Y_0^0$ as,

$$d_c = \frac{1}{\sqrt{\pi}} \int r(\theta, \phi) Y_0^0(\theta, \phi) d\Omega \tag{10}$$

Where $r(\theta, \phi)$ is the surface of the membrane. The mean diameter, was obtained by

averaging over 6 independent simulations and saving the state of the membrane 4000 times by simulation. The resulting value was $d_c \approx 800$ Å, the experimental value obtained by using cryo-electron tomography [5] varies between 650 Å and 930 Å[6].

The bending rigidity of the membranes in the simulation has been determined using the Helfrich theory as described in [7]. The free energy associated with the shape of the membrane surface can be expressed in terns of the projection of the surface onto the spherical harmonics as,

$$E = \frac{k_c}{2r_0^2} \sum_{l,m} |a_{lm}|^2 [l(l+2)(l^2+1)] \tag{11}$$

where $r_0 = d_c/2$ is the radius of the membrane and a_{lm} is the projection of the surface onto the spherical harmonic Y_l^m,

$$a_{lm} = \int r(\theta, \phi) Y_l^m(\theta, \phi) d\Omega \tag{12}$$

Using statistical mechanics it can be show that the variance of the amplitudes a_l, is related with the bending rigidity as,

$$\langle a_l^2 \rangle = \frac{r_0^2 k_B T}{k_c} [l(l+2)(l^2-1)]^{-1} \tag{13}$$

Where,

$$\langle a_l^2 \rangle = \frac{1}{2l+1} \sum_{|m|\langle l} \langle a_{lm}^2 \rangle \tag{14}$$

We have obtained the values of $\langle a_{lm}^2 \rangle$ averaging over 6 independent simulations and 4000 simulation steps, and the value of k_c has been obtained fitting the results to a function of the form,

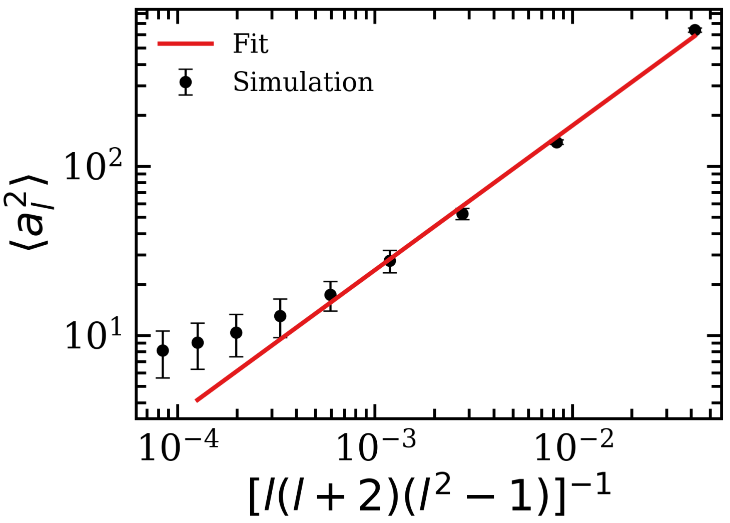

Figure S9: $\langle a_l^2 \rangle$ as a function of $l(l+2)(l^2-1)$ obtained averaging over 6 independent simulations and fit of the results using equation 15. The fitting for high values of l is not accurate because beads are too big to solve accurately the shape fluctuations for values of $l \geq 7$

$$\ln(\langle a_l^2 \rangle) = b - m \ \ln[l(l+2)(l^2-1)] \tag{15}$$

With $b = \ln(2r_0^2 k_B T/k_c)$ and $m \approx 1$.

From the fit of figure S9 we have obtained that the bending rigidity in the simulations is $k_c \approx 39 k_B T$. It is a typical value for lipidic membranes, and it leads to a membrane with relatively strong deformations. In cryo-TEM images it has also been observed that the membrane can have big deformations [5, 8].

Finally, the 2D diffusion coefficient of the beads in the membrane has been characterized computing the 2D mean square displacement of the beads in a planar membrane with small fluctuations in the z direction. The value of the diffusion coefficient of the beads in the membrane is $D_{2D} \approx 0.94$ Å. This value is in the range of values observed in

[9].

Spikes and membrane coupling

In this section, we describe the methodology employed to couple the membrane model with the spike model, a key aspect of our simulation framework. This coupling was achieved by integrating the last bead of the spike model into the membrane, representing the region of the spike that is embedded in the membrane. Consequently, this bead assumes a dual character: it is part of both the spike and the membrane.

The integration process involves the strategic placement of all spikes (a total of 30)[6] onto the membrane, ensuring that there are no collisions during the initialization phase.

For the interactions between different spikes, as well as between the spikes and the membrane, we employ a Weeks-Chandler-Andersen (WCA) potential. This choice is based on the current understanding that there are no significant interactions between the spikes or between the spikes and the membrane beyond the region of the spike embedded within the membrane [4]. The WCA potential effectively models these interactions by preventing overlap between the spikes and between the spikes and the membrane, reflecting the physical exclusion without specifying detailed interaction forces.

This approach to coupling provides a realistic and computationally efficient representation of the virus structure. It allows us to simulate the behavior of the spikes in relation to the membrane accurately, which is crucial for understanding the overall adhesion process of the virus to surfaces. The dual nature of the last bead of each spike plays a pivotal role in this model, capturing the essential interface between the spike and the membrane.

In conclusion, our model, comprising approximately 2000 beads, strikes an optimal balance between detail and computational efficiency. This allows us to conduct the ex-

tensive umbrella sampling necessary for our study, effectively handling the large number of simulations required to explore the virus adhesion process in depth.

Surface interaction

In our model's concluding section, we elaborate on the interactions between the viral membrane, the spike proteins, and the surface. A feature of our model is that the shape of the interaction potential is the same for both the membrane and spike protein beads, though the specific parameters may vary.

The interaction between each bead and the surface is governed by a Lennard-Jones-like potential. The potential $U(\Delta z)$, as a function of the distance $\Delta z = z - z_0$ (with z being the z-coordinate of the bead and z_0 the position of the surface), is expressed as follows:

For beads where the interaction energy parameter ε is negative, indicating repulsion, the potential is:

$$U(\Delta z) = \begin{cases} -4\varepsilon \left[\left(\frac{\sigma}{\Delta z}\right)^{12} - \left(\frac{\sigma}{\Delta z}\right)^{6} \right] - \varepsilon, & \text{if } \Delta z \leq 2^{1/6}\sigma, \\ 0, & \text{if } \Delta z > 2^{1/6}\sigma \end{cases} \tag{16}$$

In this scenario, the potential mimics a purely repulsive force akin to the WCA potential, operational up to a cutoff distance $z_{cut} = 2^{1/6}\sigma$, beyond which the potential is set to zero.

Conversely, for beads where ε is positive, indicative of attraction, the potential has an attractive component similar to the standard Lennard-Jones potential:

$$U(\Delta z) = -4\varepsilon \left[\left(\frac{\sigma}{\Delta z}\right)^{12} - 2\left(\frac{\sigma}{\Delta z}\right)^{6} \right] \tag{17}$$

Here, σ is the parameter associated with the size of the bead, which can differ for membrane and spike protein beads. This dual application of the potential allows for both attractive and repulsive interactions, tailored by the specific values of ε and σ for each type of bead.

Videos of CG virus model

Videos in Supplementary Added Material illustrate the dynamics of the virus adhesion, including the envelope deformation and the spike diffusion over the membrane.

- Video 1: Mild spike-substrate interaction and strong membrane-substrate interaction: $\varepsilon_{\text{spk}} = 1.0$ and $\varepsilon_{\text{mem}} = 1.2$
- Video 2: Similar spike-substrate and membrane-substrate interactions: $\varepsilon_{\text{spk}} = 3$ and $\varepsilon_{\text{mem}} = 1.2$
- Video 3: Strong spike-substrate interaction and mild membrane-substrate interaction: $\varepsilon_{\text{spk}} = 3.5$ and $\varepsilon_{\text{mem}} = 0.8$
- Video 4: Free coronavirus (not adsorbed).

References

[1] Hongyan Yuan, Changjin Huang, Ju Li, George Lykotrafitis, and Sulin Zhang. One-particle-thick, solvent-free, coarse-grained model for biological and biomimetic fluid membranes. *Phys. Rev. E*, 82:011905, Jul 2010.

[2] Timothy Leong, Chandhana Voleti, and Zhangli Peng. Coarse-grained modeling of coronavirus spike proteins and ace2 receptors. *Frontiers in Physics*, 9, 2021.

[3] Anton Arkhipov, Peter L. Freddolino, and Klaus Schulten. Stability and dynamics of virus capsids described by coarse-grained modeling. *Structure*, 14(12):1767–1777, 2006.

[4] et al. Zunlong Ze. Structures and distributions of SARS-CoV-2 spike proteins on intact virions. *Nature*, 588:498–502, 2020.

[5] Linhua Tai, Guoliang Zhu, Minnan Yang, Lei Cao, Xiaorui Xing, Guoliang Yin, Chun Chan, Cheng-Feng Qin, Zihe Rao, Xiangxi Wang, Fei Sun, and Yun Zhu. Nanometer-resolution in situ structure of the SARS-Cov-2 postfusion spike protein. *Proceedings of the National Academy of Sciences*, 118:e2112703118, 11 2021.

[6] Montserrat Bárcena, Gert T. Oostergetel, Willem Bartelink, Frank G. A. Faas, Arie Verkleij, Peter J. M. Rottier, Abraham J. Koster, and Berend Jan Bosch. Cryo-electron tomography of mouse hepatitis virus: Insights into the structure of the coronavirion. *Proceedings of the National Academy of Sciences*, 106(2):582–587, 2009.

[7] David A. Rower and Paul J. Atzberger. Heterogeneous vesicles with phases having different preferred curvatures: Shape fluctuations and mechanics of active deformations, 2019.

[8] Beata Turonova, Mateusz Sikora, Christoph Schürmann, Wim Hagen, Sonja Welsch, Florian Blanc, Sören Bülow, Michael Gecht, Katrin Bagola, Cindy Hörner, Ger Zandbergen, Jonathan Landry, Nayara Azevedo, Shyamal Mosalaganti, Andre Schwarz, Roberto Covino, Michael Mühlebach, Gerhard Hummer, Jacomine Locker, and Martin Beck. In situ structural analysis of SARS-Cov-2 spike reveals flexibility mediated by three hinges. *Science*, 370:eabd5223, 08 2020.

[9] Beibei Wang, Changqing Zhong, and D Tieleman. Supramolecular organization of SARS-Cov and SARS-Cov-2 virions revealed by coarse-grained models of intact virus envelopes. *Journal of Chemical Information and Modeling*, 62, 12 2021.
